# Defining the ionic mechanisms of optogenetic control of vascular tone by channelrhodopsin‐2

**DOI:** 10.1111/bph.14183

**Published:** 2018-04-17

**Authors:** Nils J G Rorsman, Chau M Ta, Hannah Garnett, Pawel Swietach, Paolo Tammaro

**Affiliations:** ^1^ Department of Pharmacology University of Oxford Oxford UK; ^2^ OXION Initiative in Ion Channels and Disease University of Oxford Oxford UK; ^3^ Department of Physiology, Anatomy and Genetics University of Oxford Oxford UK

## Abstract

**Background and Purpose:**

Optogenetic control of electromechanical coupling in vascular smooth muscle cells (VSMCs) is emerging as a powerful research tool with potential applications in drug discovery and therapeutics. However, the precise ionic mechanisms involved in this control remain unclear.

**Experimental Approach:**

Cell imaging, patch‐clamp electrophysiology and muscle tension recordings were used to define these mechanisms over a wide range of light stimulations.

**Key Results:**

Transgenic mice expressing a channelrhodopsin‐2 variant [ChR2(H134R)] selectively in VSMCs were generated. Isolated VSMCs obtained from these mice demonstrated blue light‐induced depolarizing whole‐cell currents. Fine control of artery tone was attained by varying the intensity of the light stimulus. This arterial response was sufficient to overcome the endogenous, melanopsin‐mediated, light‐evoked, arterial relaxation observed in the presence of contractile agonists. Ca^2+^ entry through voltage‐gated Ca^2+^ channels, and opening of plasmalemmal depolarizing channels (TMEM16A and TRPM) and intracellular IP_3_ receptors were involved in the ChR2(H134R)‐dependent arterial response to blue light at intensities lower than ~0.1 mW·mm^−2^. Light stimuli of greater intensity evoked a significant Ca^2+^ influx directly through ChR2(H134R) and produced marked intracellular alkalinization of VSMCs.

**Conclusions and Implications:**

We identified the range of light intensity allowing optical control of arterial tone, primarily by means of endogenous channels and without substantial alteration to intracellular pH. Within this range, mice expressing ChR2(H134R) in VSMCs are a powerful experimental model for achieving accurate and tuneable optical voltage‐clamp of VSMCs and finely graded control of arterial tone, offering new approaches to the discovery of vasorelaxant drugs.

**Abbreviations**
2*‐*APB: 2‐aminoethoxydiphenyl borate
Ani9: 2‐(4‐Chloro‐2‐methylphenoxy)‐N'‐(2‐methoxybenzylidene) acetohydrazide
CaCC: Ca^2+^‐activated Cl^−^ channel
Ca_v_: voltage‐gated Ca^2^^+^ channel
ChR2: channelrhodopsin‐2
ChR2(H134R): channelrhodopsin‐2 in which histidine at position 134 was substituted with an arginine
CICR: Ca^2+^‐induced Ca^2+^ release
EC: endothelial cell
eYFP: enhanced yellow fluorescent protein
MONNA: 2‐[(4‐Methoxy‐2‐naphthalenyl)amino]‐5‐nitro‐benzoic acid
pH_i_: intracellular pH
RyR: ryanodine receptor
*V*_*m*_: cell membrane potential
VSMC: vascular smooth muscle cell

## Introduction

Optogenetics is an experimental approach involving the use of light in conjunction with microbial opsins (light‐sensitive proteins) to control cell, tissue or organ function (Fenno *et al*., 2011; Lin, 2011; Entcheva, 2013). For instance, cells engineered to express the non‐selective, blue light‐activated cation channel channelrhodopsin‐2 (ChR2) can be controlled for excitability with exceptional spatio‐temporal accuracy (Fenno *et al*., 2011; Lin, 2011; Entcheva, 2013). The ChR2 channel is permeable to monovalent and divalent cations, including H^+^, Na^+^ and Ca^2+^. Thus, opening of ChR2 channels typically leads to cell membrane potential (*V*_*m*_) depolarization (Nagel *et al*., 2003; Caldwell *et al*., 2008; Lin, 2011). Several ChR2 variants have been generated, including ChR2(H134R), characterized by reduced current desensitization and enhanced photocurrents as a consequence of the replacement of histidine at position 134 by arginine (Nagel *et al*., 2005; Lin, 2011; Reinbothe *et al*., 2014; Bruegmann *et al*., 2015; Wu *et al*., 2015).

ChR2 and its variants have been used extensively as experimental tools in the field of neuroscience to define brain circuitry (Boyden *et al*., 2005; Zhang *et al*., 2007; Yizhar *et al*., 2011). These opsins have also been harnessed to control the function of non‐neuronal cell types. For example, ChR2 has enabled control of myocardial excitability and inotropy (Arrenberg *et al*., 2010; Bruegmann *et al*., 2010; Entcheva, 2013) and skeletal muscle contraction (Asano *et al*., 2012; Sakar *et al*., 2012; Bruegmann *et al*., 2015). Optogenetics has also the potential to revolutionize vascular research. An advance in this direction was recently made by Wu *et al*. (2015), who selectively expressed a ChR2 mutant in murine vascular smooth muscle cells (VSMCs) to achieve optogenetic control of VSMC contraction and hence arterial tone. In their study, isolated aortic VSMCs expressing ChR2 channels demonstrated blue light‐induced currents. The tone of isolated mesenteric artery rings increased in response to high‐intensity blue light stimuli (ranging from 10 to 24 mW·mm^−2^), which were reported to evoke maximal ChR2 channel activation (Lin *et al*., 2009; Mattis *et al*., 2011). Furthermore, it was possible to optogenetically modulate the perfusion pressure of the coronary and renal circulations in isolated hearts and kidneys (Wu *et al*., 2015). Optogenetics has also been used to gain insights into the role of arterioles and pericyte‐covered capillaries in the control of cerebral blood flow (Hill *et al*., 2015). These important studies have demonstrated the utility and potential of optogenetics in vascular research. Critically important mechanistic aspects, however, need to be addressed to enable a complete interpretation of vascular optogenetics experiments and to exploit this technology in an *in vivo* setting. This technology has the potential of offering new approaches to the identification and testing of new compounds that could relieve vasoconstriction.

To provide a more complete and comprehensive mechanistic appraisal of vascular optogenetics, it is critical to probe arterial responses over an adequately wide range of light stimulations to determine whether different degrees of ChR2 activation produce fine, graded control of vessel tone. Furthermore, it is well established that isolated wild‐type mammalian systemic arteries pre‐constricted with agonists, such as noradrenaline or phenylephrine, relax in response to blue light in a ChR2‐independent manner (Furchgott *et al*., 1955; Ehrreich and Furchgott, 1968; Sikka *et al*., 2014). Blue light‐mediated relaxation was also observed in murine cerebral arterioles *in situ* (Rungta *et al*., 2017). Thus, evaluating the capacity of ChR2‐induced contraction to overcome the intrinsic blue light‐dependent arterial relaxation is an essential prerequisite for establishing vascular optogenetics *in vivo*, where the circulating levels of catecholamines will vary depending on the extent of the sympathetic drive. It is also important to explain the precise ionic mechanisms linking light stimulation with arterial contraction. This includes an understanding of the extent to which ChR2 channel opening evokes depolarization and activates endogenous channels, as opposed to providing a direct route for non‐physiological Ca^2+^ entry. A related point is to assess possible changes in intracellular pH (pH_i_) of VSMCs, which may occur as a consequence of prolonged ChR2 activation due to its permeability to H^+^ (Nagel *et al*., 2003; Lin *et al*., 2009; Berndt *et al*., 2010, 2011; Gradmann *et al*., 2011). Light‐evoked changes in pH_i_ would be highly relevant because of their known modulatory effects on of excitation–contraction coupling and responses to pharmacological agents (Wakabayashi *et al*., 2006; Boedtkjer and Aalkjaer, 2012).

To address the issues outlined above, we generated mice expressing ChR2(H134R) selectively in VSMCs. We systematically examined the effects of a wide range (0.01–12.1 mW·mm^−2^) of blue light intensities on both the magnitude of ChR2(H134R) current in isolated VSMCs and the extent of contraction of artery rings. A broad range of artery type was included in these studies, namely, large conduit, small systemic and pulmonary arteries. In the case of isolated aortic VSMCs and isolated rings, experiments were extended to accurately map the effect of blue light on *V*_*m*_ and on the ionic mechanisms involved in contraction. We found that expression of ChR2(H134R) channels enabled optical voltage clamp of these VSMCs. At blue light stimulations lower than 0.1 mW·mm^−2^, ChR2(H134R) activation served as a depolarizing trigger for endogenous voltage‐gated Ca^2+^ (http://www.guidetopharmacology.org/GRAC/FamilyIntroductionForward?familyId=80 and Ca^2+^‐activated depolarizing channels, such as the TMEM16A http://www.guidetopharmacology.org/GRAC/FamilyDisplayForward?familyId=130 (CaCCs) and http://www.guidetopharmacology.org/GRAC/FamilyDisplayForward?familyId=78 non‐selective cation channels. At higher intensities of blue light, substantial ChR2(H134R)‐mediated Ca^2+^ influx increased the likelihood of Ca^2+^ overload. Additionally, a meaningful increase of pH_i_ was observed at these higher light intensities, adding another level of influence on excitation–contraction coupling. Overall, this work provided a detailed analysis of the use of ChR2(H134R) channels in vascular optogenetics, which highlights its potential and identifies the limitations for use in vascular research, including the identification of novel vasodilating drugs.

## Methods

An expanded Methods section is available in the Supporting Information.

### Generation of ChR2(H134R)‐expressing mice

All animal care and experimental protocols were in accordance with the UK Home Office regulations (Guidance on the Operation of Animals, Scientific Procedures Act, 1986). The use of transgenic mice was approved by the University Ethics Committee. Animal studies are reported in compliance with the ARRIVE guidelines (Kilkenny *et al*., 2010; McGrath and Lilley, 2015).

Mice expressing ChR2(H134R) tagged with the enhanced yellow fluorescent protein [ChR2(H134R)‐eYFP] specifically in smooth muscle were generated and termed ChR2(H134R)‐SM mice. Mice engineered to harbour the ChR2(H134R)‐eYFP coding sequence within the Gt(ROSA)26Sor locus downstream of a STOP cassette were used as a control. Fuller descriptions of the mouse strains used (from Jackson Laboratories) are given in the Supporting Information. Mice (maximum six per cage) were housed in a pathogen‐free environment in Tecniplast Sealsafe IVC cages (floor area 542 cm^2^). Treated Aspen chip and Sizzle‐Nest bedding and environmental enrichment (such as play tunnels) were present in each cage. Mice were maintained in a 12 h light/dark cycle, in controlled temperatures (20–22°C) and fed normal chow and water *ad libitum*. For each genotype, mice of either sex were selected randomly on the day of the experiment. Adult mice aged 8–14 weeks were used in this study.

### Cell fluorescence imaging

Confocal and epifluorescence microscopy was used to estimate the cellular distribution and degree of expression of ChR2(H134R) in freshly isolated VSMCs (aortic, mesenteric and pulmonary) respectively (Adomaviciene *et al*., 2013; McCloy *et al*., 2014).

### Electrophysiology

Ionic currents were recorded with the whole‐cell configuration of the patch‐clamp technique in isolated VSMCs. The *V*_*m*_ was recorded with the perforated patch‐clamp configuration during current clamp. Current recordings were normalized to cell capacitance to obtain current density (in pA·pF^−1^). Only cells with a seal resistance ≥1 GΩ were considered for analysis. Cells were exposed to pulses of blue light (470 nm wavelength) of 0.01–12.1 mW·mm^−2^ intensity. Duration of the pulses was 2 s, unless stated otherwise.

ChR2(H134R) currents were measured at −80 mV. The ChR2(H134R) current versus blue light intensity relationship was fitted with the Hill equation of the form:

(1) $$I=\frac{I_{\max}}{1+{\left(\frac{{\mathrm{EL}}_{50}}{\text{light intensity}}\right)}^{h}},$$

where *I* is the steady‐state ChR2(H134R) current density measured at a given light intensity, *I*_max_ is the maximal asymptotic current reached, EL_50_ is the light intensity causing half‐maximal current activation and *h* is the slope coefficient. The small current observed in the dark was subtracted offline from the current measured at each light intensity. Thus, *I* represents the ChR2(H134R) blue light‐activated current.

The membrane potential (*V*_*m*_) versus light intensity relationship was fitted with a modified Hill equation of the form:

(2) $$V_{m}=V_{\text{rest}}+\frac{V_{\max}-V_{\text{rest}}}{1+{\left(\frac{{\mathrm{EL}}_{50}}{\text{light intensity}}\right)}^{v}},$$

where *V*_*m*_ is the membrane potential, *V*_rest_ is the resting *V*_*m*_ (in the absence of light stimulation), *V*_max_ is the maximal asymptotic *V*_*m*_ reached, EL_50_ is the light intensity causing half‐maximal membrane depolarization and *v* is the slope coefficient.

The whole‐cell current density versus voltage relationship for Ca_V_ channels was obtained by applying 200 ms pulses from −70 to +60 mV in 5 mV steps from a holding potential (*V*_hold_) of −60 mV. The peak current density was measured for each *V*_*m*_ and plotted versus *V*_*m*_. The resulting relationship was fitted with a modified Boltzmann equation of the form:

(3) $$I_{p}=G_{\max}\times \frac{\left(V_{m}-V_{\mathrm{rev}}\right)}{1+\exp\left(\frac{V_{m}-V_{0.5,a}}{s_{a}}\right)},$$

where *I*_*p*_ is the peak current density, *G*_max_ is the whole‐cell Ca^2+^ conductance, *V*_rev_ is the whole‐cell current reversal potential, *V*_0.5,*a*_ is the midpoint voltage for current activation and *s*_*a*_ is the *e*‐fold slope.

Steady‐state inactivation properties were assessed by applying 1.5 s conditioning prepulses from −90 to +40 mV in 10 mV steps. Each prepulse was followed by a 200 ms test pulse to +10 mV. The *V*_hold_ was −60 mV. The peak current (*I*_*p*_) in response to each test pulse was measured, normalized for the maximal *I*_*p*_ and plotted versus the *V*_*m*_ of the prepulse. The resulting steady‐state inactivation was fitted with a Boltzmann equation of the form:

(4) $$I_{p}=\frac{I_{\max}}{1+\exp\left(\frac{V_{m}-V_{0.5,h}}{s_{h}}\right)},$$

where *I*_max_ is the maximal current and *V*_0.5,*h*_ is the midpoint voltage for current inactivation and *s*_*h*_ is the *e*‐fold slope.

### Myography

Contraction of isolated artery (aortic, mesenteric and pulmonary) rings was measured using a small vessel wire myograph (model 420A, Danish Myo Technology, Aarhus, Denmark). Tension measurements (in mN) were normalized for the artery ring length (in mm) to enable comparison of data obtained from rings of slightly different length. Tissue was stimulated with blue light of various intensities (0.02–7.21 mW·mm^−2^). Duration of the pulses was 7 min, unless stated otherwise.

The tension versus blue light intensity relationships in the absence of PE were fitted with the Hill equation of the form:

(5) $$T=\frac{T_{\mathrm{ss}}}{1+{\left(\frac{{\mathrm{EL}}_{50}}{\text{light intensity}}\right)}^{s}},$$

where *T* is the tension (normalized for length of the artery ring) measured at a given light intensity, *T*_*ss*_ is the maximal asymptotic tension reached, EL_50_ is the light intensity causing half‐maximal tension and *s* is the slope coefficient.

As outlined in the main text and shown in Figure 7, the response of isolated aortic rings to phenylephrine was characterized by a rapid contraction followed by a transient relaxation and a slow increase in tension. Thus, phenylephrine did not induce a stable level of contraction. To account for this changing level of tension, in the experiments conducted in the presence of phenylephrine (Figure 4), the effect of each light stimulation on artery ring tension was expressed relative to the mean of the tension measured immediately before and after a given light exposure and the average data fitted using Equation (5).

Equation (5) was also used to quantify the tension versus extracellular KCl concentration ([KCl]_e_) (Supporting Information Figure S1) or noradrenaline concentration ([NA]) (Supporting Information Figure S3) relationships. In these cases, the independent variable was [KCl]_e_ or [NA], and EL_50_ was substituted with EC_50_, the [KCl]_e_ or [NA] causing half‐maximal response.

### pH imaging

Freshly isolated aortic VSMCs were loaded with cSNARF1 (10 μM of its AM‐ester for 10 min) and imaged for pH ratiometrically (Zeiss LSM confocal system; excitation 555 nm, emission collected simultaneously at 580 and 640 nm). Ratio was converted to pH_i_ using a calibration curve determined in separate experiments by nigericin clamping of pH_i_. pH_i_ was measured before and after 7 min exposure of blue light of various intensities (0.03–2.1 mW·mm^−2^).

### Blue light stimulation systems for optogenetics

In patch‐clamp experiments, optical stimulation of ChR2(H134R)‐expressing VSMCs was performed using an optoLED system (Cairn Research, Kent, UK), comprising of a 3.5 W blue light (470 nm)‐emitting diode (LED) mounted on the rear port of an inverted microscope (IX51, Olympus, Southend‐on‐Sea, UK). The optoLED system was controlled by GePulse software (Dr M. Pusch, CNR, Italy) with an analogue‐to‐digital and digital‐to‐analogue converter (USB‐6221; National Instruments, Newbury, UK).

For myography experiments, blue light was provided by a 5 W LED, controlled by a power supply made in‐house (Mr M. Preston, Dept. Pharmacology, University of Oxford) and delivered through a glass window positioned at the bottom of the myography bath chambers. Intensity and duration of the stimulus were digitally controlled. In pH imaging experiments, the same LED power supply system was used, and blue light was delivered to the sample through a liquid light guide with a core diameter of 3.0 mm (Newport, Irvine, CA, USA), reaching the sample on the microscope stage perpendicularly from above.

Light intensity (in mW·mm^−2^) was measured at the sample (cell or tissue) site using a Newport digital optical power meter (Model 843, Newport), with a sensor area of 0.4 × 0.4 cm, with wavelength set at 470 nm. Calibration performed at the beginning of the experiments confirmed that the LED current (in the range 0–1.0 A) was linearly related to the illumination wattage at the sample plane.

### Blinding

It was not feasible for the operator to be blinded to the genotype of the mice used during each individual experiment as the absence or presence of the effects assessed (such as the ionic current or force of contraction), immediately revealed the genotype of the sample under examination. The methods of analysis were established during study design, and prior to execution of the experiments, to remove possible operator bias.

### Data and statistical analysis

The data and statistical analysis in this study comply with the recommendations on experimental design and analysis in pharmacology (Curtis *et al*., 2015). Multiple measurements on samples derived from a single animal (such as isolated cells or vessel fragments) were considered as non‐independent samples. Thus, we averaged the results per animal to obtain a single value. Statistical analyses were therefore carried out on the complete data set of independent experiments. The number of animals used was ≥5 in each case. Results are expressed as mean ± SEM of *N* (number of independent experiments, i.e. number of animals). The number of total observations (i.e. numbers of vessels or cells) is reported in the figure legends and tables, and it is denoted by the letter *n*. Statistical significance was determined with two‐tailed unpaired *t*‐test or one‐way ANOVA with Bonferroni's *post hoc* test, as appropriate. For all statistical tests, *P*‐values <0.05 were considered significant. SPSS (version 22; SPSS Inc., Chicago, IL, USA) or Excel (2013 Edition, Microsoft, Redmond, WA, USA) were used for statistical analysis.

### Materials

Compounds used in the experiments described here were supplied as follows: nifedipine, noradrenaline, phenylephrine and http://www.guidetopharmacology.org/GRAC/LigandDisplayForward?ligandId=4303 by Sigma*‐*Aldrich (Gillingham, Dorset, UK); http://www.guidetopharmacology.org/GRAC/LigandDisplayForward?ligandId=2433), Ani9, http://www.guidetopharmacology.org/GRAC/LigandDisplayForward?ligandId=2330 and MONNA by Tocris (Abingdon, Oxfordshire, UK).

### Nomenclature of targets and ligands

Key protein targets and ligands in this article are hyperlinked to corresponding entries in http://www.guidetopharmacology.org/, the common portal for data from the IUPHAR/BPS Guide to PHARMACOLOGY (Harding *et al*., 2018), and are permanently archived in the Concise Guide to PHARMACOLOGY 2017/18 (Alexander *et al*., 2017a,b,c).

## Results

### ChR2(H134R) expression and current density in VSMCs

**Figure 1:**
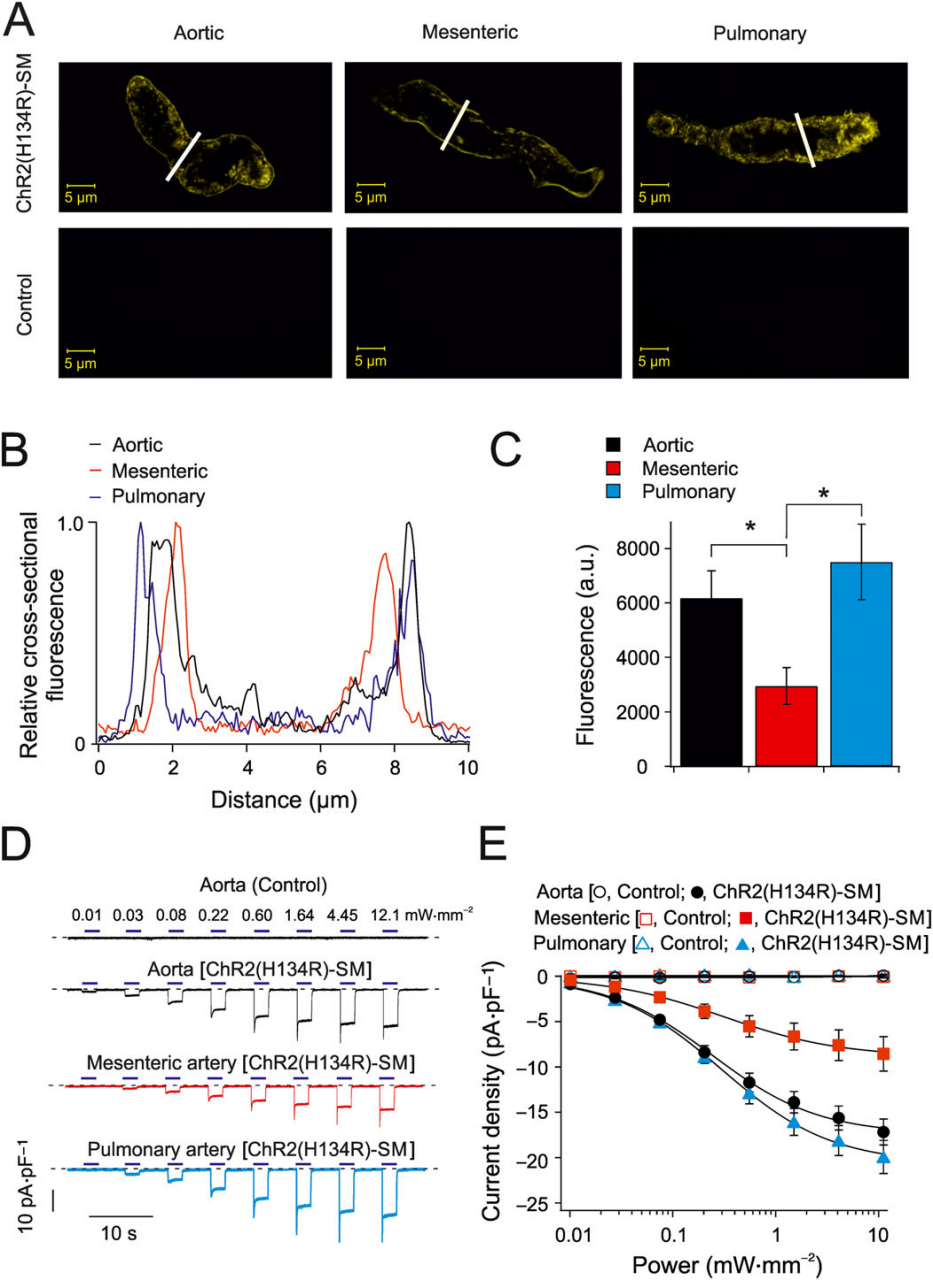
Expression of ChR2(H134R) channels and current density in isolated VSMCs. (A) Representative confocal images of freshly isolated aortic, mesenteric and pulmonary VSMCs obtained from control and ChR2(H134R)‐SM mice, as indicated. Similar fluorescence distribution was observed in cells obtained from a total of five mice. (B) Normalized fluorescence intensity of cell cross sections of aortic, mesenteric and pulmonary VSMCs (cross‐section location indicated by white bars in A). (C) Average fluorescence density of aortic (*N* = 5, *n* = 61), mesenteric (*N* = 5, *n* = 49) and pulmonary artery (*N* = 5, *n* = 64) VSMCs isolated from ChR2(H134R)‐SM. (D) Whole‐cell currents recorded from aortic, mesenteric and pulmonary artery VSMCs isolated from control and ChR2(H134R)‐SM mice, as indicated. Currents were recorded at −80 mV. VSMCs isolated from control and ChR2(H134R)‐SM mice were exposed to periods (2 s) of blue light of various intensities, as indicated by the horizontal blue bars. The dashed lines represent the level of current in the absence of light stimulation. (E) Mean current density versus blue light intensity relationships for VSMCs obtained from control aortic (*N* = 5, *n* = 5), mesenteric (*N* = 5, *n* = 10) and pulmonary artery (*N* = 5, *n* = 6) VSMCs or ChR2(H134R)‐SM aortic (*N* = 10, *n* = 18), mesenteric (*N* = 6, *n* = 16) and pulmonary artery (*N* = 7, *n* = 23) VSMCs. The curves through the open symbols (control) were drawn by the eye. The curves through the filled symbols represent the best fit of the data with Equation (1). ^*^ *P* < 0.05, significantly different as indicated; one‐way ANOVA.

To assess the utility of vascular optogenetics in different vascular beds, we determined the degree of ChR2(H134R) expression in various artery types obtained from ChR2(H134R)‐SM mice, as this may influence the arterial sensitivity to blue light stimuli. VSMCs (from aortic, mesenteric and pulmonary arteries) were isolated from control and ChR2(H134R)‐SM mice and imaged confocally to assess ChR2(H134R) distribution within the cell (Figure 1A–C). VSMCs obtained from ChR2(H134R)‐SM mice had prominent eYFP signal demonstrating expression of the channel construct (Figure 1A). The fluorescence signal was primarily detected in the plasma membrane of VSMCs (Figure 1B), as indicated by the eYFP intensity profiles (along the solid white lines in Figure 1A). In contrast, no detectable fluorescence was identified in VSMCs isolated from control mice (Figure 1A). To obtain a measure of the total ChR2(H134R) expression in the various VSMCs types, the average eYFP fluorescence intensity was assessed by epifluorescence microscopy and normalized for cell area (fluorescence density). The fluorescence density in mesenteric VSMCs was about ~0.5 that of the aortic VSMCs, while the intensity of pulmonary artery VSMCs was indistinguishable from that of the aortic VSMCs (Figure 1C).

To test if differences in ChR2(H134R) expression affected the current density, whole‐cell currents were recorded from aortic, mesenteric and pulmonary VSMCs isolated from ChR2(H134R)‐SM mice. As observed with ChR2(H134R) currents in heterologous expression systems (Nagel *et al*., 2005; Lin *et al*., 2009; Berndt *et al*., 2011), ChR2(H134R) currents in isolated VSMCs were characterized by a rapid activation followed by a rapid inactivation to a new steady‐state level (Figure 1D). The steady‐state current increased as light intensity was increased. The fit of the steady‐state current density versus blue light intensity relationships with Equation (1) yielded a maximal asymptotic current (*I*_max_) of approximately −18, −9 and −22 pA·pF^−1^ for aortic, mesenteric and pulmonary VSMCs respectively (Table 1). The light intensity causing half‐maximal effect (EL_50_) was ~0.3 mW·mm^−2^, and the slope factor (*h*) was ~0.6–0.8 in each case (Table 1). Thus, different expression levels of ChR2(H134R) resulted in currents with different amplitudes (*I*_max_), but of similar sensitivity to blue light, quantified in terms of parameters EL_50_ and *h*. In contrast, there was essentially no current increase observed in VSMCs obtained from control mice. For instance, the current in the presence of 12.1 mW·mm^−2^ blue light was 0.1 ± 0.2 pA·pF^−1^ (*N* = 5, aortic), 0.1 ± 0.1 pA·pF^−1^ (*N* = 5, mesenteric) and 0.1 ± 0.1 pA·pF^−1^ (*N* = 5, pulmonary) in VSMCs obtained from control mice.

**Table 1:** Parameters for the current versus blue light intensity relationships for VSMCs obtained from ChR2(H134R)‐SM mice

	ChR2(H134R)‐SM mice		
	Aorta	Mesenteric artery	Pulmonary artery
EL_50_ (mW·mm^−2^)	0.31 ± 0.03 (*N* = 10, *n* = 18)	0.32 ± 0.02 (*N* = 6, *n* = 16)	0.37 ± 0.04 (*N* = 7, *n* = 23)
*h*	0.8 ± 0.1 (*N* = 10, *n* = 18)	0.6 ± 0.1 (*N* = 6, *n* = 16)	0.8 ± 0.1 (*N* = 7, *n* = 23)
*I*_max_ (pA·pF^−1^)	–18 ± 1 (*N* = 10, *n* = 18)	–9 ± 2 (*N* = 6, *n* = 16)	−22 ± 2 (*N* = 7, *n* = 23)

Parameters (EL_50_, *h* and *I*_max_) obtained from the Hill fit (Equation (1)) of the current versus light intensity relationship for aortic, pulmonary and mesenteric VSMCs obtained from ChR2(H134R)‐SM mice.

### Relationship between blue light intensity and tension in isolated artery rings

**Figure 2:**
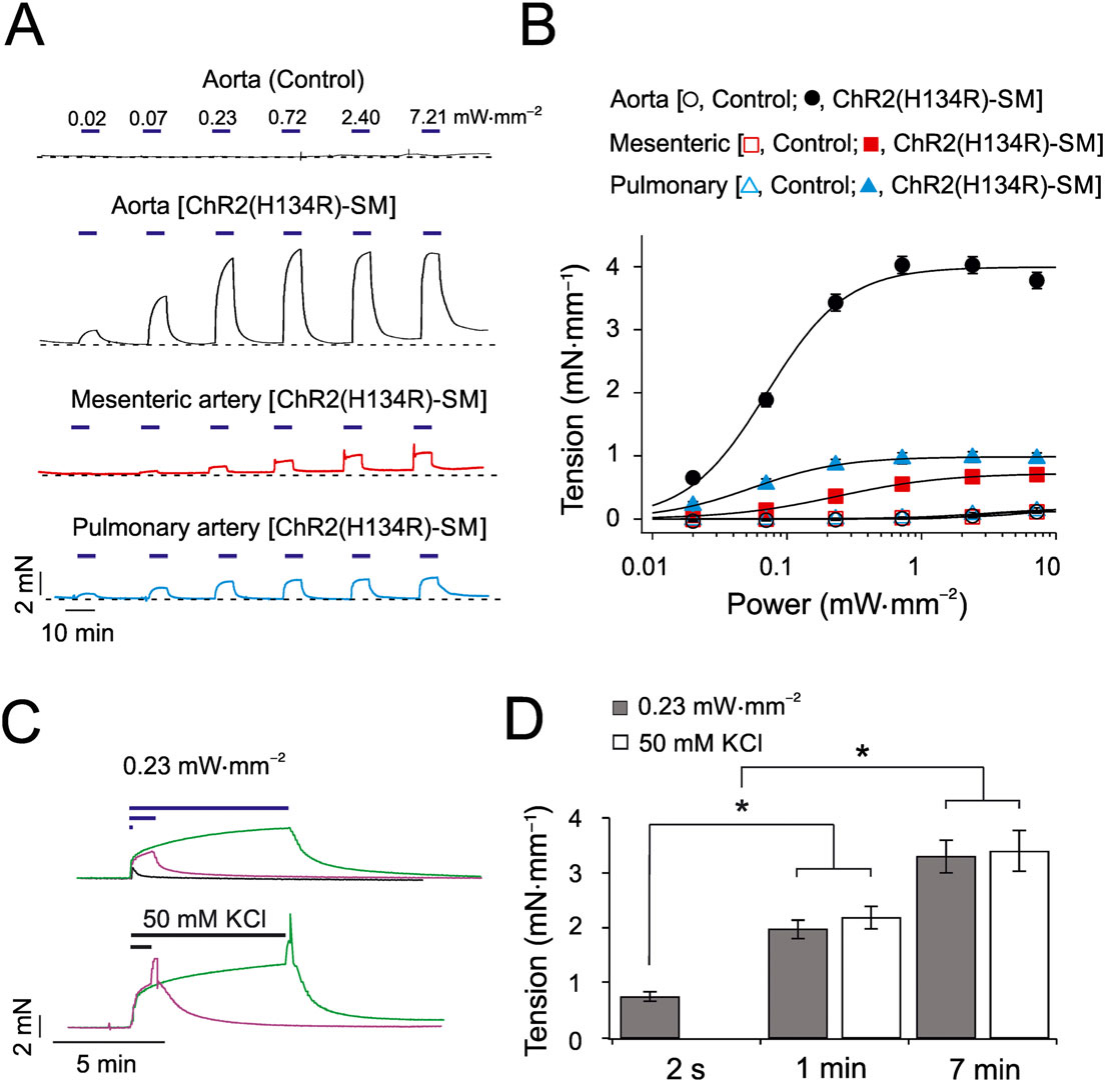
Effects of blue light on the tension of isolated artery rings. (A) Isometric tension recordings obtained from aortic, mesenteric and pulmonary artery rings isolated from control and ChR2(H134R)‐SM mice, as indicated. Artery rings were exposed to periods (7 min) of blue light of various intensities, as indicated by horizontal blue bars. Dashed lines represent baseline tension level. (B) Mean tension versus blue light intensity relationships for (i) control aortic (*N* = 5, *n* = 10), mesenteric (*N* = 5, *n* = 6) and pulmonary artery (*N* = 5, *n* = 6) rings or (ii) aortic (*N* = 32, *n* = 36), mesenteric (*N* = 10, *n* = 15) and pulmonary artery (*N* = 5, *n* = 9) artery rings obtained from ChR2(H134R)‐SM mice, as indicated. The curves through open symbols (control) were drawn by the eye. The curves through the filled symbols represent the best fit of the data with Equation (5). (C) Isometric tension recordings obtained from isolated aortic rings in response to periods of exposure to blue light (0.23 mW·mm^−2^) of different durations (2 s, 1 min and 7 min) or periods of exposure (1 or 7 min) to PSS supplemented with 50 mM KCl, as indicated by the horizontal bars. Traces are represented in different colours for clarity. (D) Mean tension developed by isolated aortic rings in response to 0.23 mW·mm^−2^ blue light or 50 mM KCl for periods of different duration (*N* = 5, *n* = 10 in each case). ^*^ *P* < 0.05, significantly different as indicated; one‐way ANOVA.

To examine the precise relationship between the ChR2(H134R) current amplitude and changes in arterial tone, isometric tension measurements were performed in isolated aortic, mesenteric and pulmonary artery rings obtained from ChR2(H134R)‐SM mice. Isolated artery rings were exposed to periods (7 min) of blue light illumination of various intensities. The tension of the arterial rings increased as the blue light intensity increased (Figure 2A, B). The maximal level of contraction was reached in blue light intensities of >0.7 mW·mm^−2^ in aortic and pulmonary artery rings, whereas the mesenteric artery rings required blue light >2 mW·mm^−2^ to achieve maximal contraction. The fit of the tension versus blue light intensity relationships with Equation (5) yielded maximal tension (*T*_*ss*_) of ~4.0, ~0.8 and ~1.0 mN·mm^−1^ for aortic, mesenteric and pulmonary artery rings respectively. The EL_50_ was ~0.1–0.3 mW·mm^−2^, and the slope factor (*s*) was ~1.1–1.5 in each case (Table 2). Light stimulation produced a very small increase in tension in artery rings obtained from control mice, as the contraction measured in the highest blue light intensity stimulation (7.2 mW·mm^−2^) was 0.1 ± 0.1 mN·mm^−1^ (*N* = 5) for control aortic, mesenteric and pulmonary artery rings.

**Table 2:** Parameters for the tension versus blue light intensity relationships for artery rings obtained from ChR2(H134R)‐SM mice

	ChR2(H134R)‐SM mice		
	Aorta	Mesenteric artery	Pulmonary artery
EL_50_ (mW·mm^−2^)	0.08 ± 0.01 (*N* = 32, *n* = 36)	0.25 ± 0.05 (*N* = 10, *n* = 15)	0.06 ± 0.01 (*N* = 5, *n* = 9)
*s*	1.5 ± 0.1 (*N* = 32, *n* = 36)	1.1 ± 0.1 (*N* = 10, *n* = 15)	1.4 ± 0.1 (*N* = 5, *n* = 9)
*T*_*ss*_ (mN·mm^−1^)	4.0 ± 0.1 (*N* = 32, *n* = 36)	0.8 ± 0.1 (*N* = 10, *n* = 15)	1.0 ± 0.1 (*N* = 5, *n* = 9)

Parameters (EL_50_, *s* and *T*_max_) obtained from the Hill fit (Equation (5)) of the tension versus blue light intensity relationship for aortic, mesenteric and pulmonary artery rings obtained from ChR2(H134R)‐SM mice.

We next compared the extent of blue light‐induced contraction with the contraction triggered by elevation of [KCl]_e_ up to 100 mM. The maximal level of blue light‐induced contraction for aortic, mesenteric and pulmonary artery rings obtained from ChR2(H134R)‐SM mice was ~105, ~70 and ~105% of the maximal contraction obtained in response to high [KCl]_e_ (Supporting Information Figure S1). Thus, all artery types tested demonstrated maximal or near‐maximal tension at the highest levels of ChR2(H134R) activation.

### Relationship between duration of blue light exposure and tension of isolated artery rings

Experiments shown in Figure 2A illustrate that prolonged exposure (7 min) of isolated artery rings to blue light produced stable contractions. We next tested if shorter pulses of blue light could initiate contraction of isolated artery rings or whether the blue light stimulation had to be maintained in order to achieve steady‐state contraction. Aortic rings isolated from ChR2(H134R)‐SM mice were exposed to light of 0.23 mW·mm^−2^ for various time periods (Figure 2C). This light intensity was expected to shift the *V*_*m*_ to approximately −25 mV, based on the current‐clamp recordings in isolated aortic VSMCs described below. The tension of ChR2(H134R)‐SM aortic rings increased rapidly in response to light stimulation, but removal of the stimulus resulted in an immediate onset of relaxation. Light of 0.23 mW·mm^−2^ applied for 2 s, 1 min or 7 min resulted in an increase in tension of 0.7 ± 0.1 mN·mm^−1^ (*N* = 5), 2.0 ± 0.2 mN·mm^−1^ (*N* = 5) and 3.3 ± 0.3 mN·mm^−1^ (*N* = 5) respectively (Figure 2D).

To determine if the ChR2(H134R)‐mediated contraction was directly linked to *V*_*m*_ depolarization, we compared the extent of contraction caused by ChR2(H134R) opening with the contraction caused by elevation of [KCl]_e_. The solution was supplemented with 50 mM KCl, as this was expected to produce *V*_*m*_ depolarization to approximately −25 mV (Nelson and Quayle, 1995; Aidley, 1998). Aortic rings were exposed to elevated [KCl]_e_ for 1 or 7 min. Note that, in these experiments, solution exchange in the myography chamber required ~1 s and thus, KCl application for 2 s was not attempted. Application of 50 mM KCl for 1 or 7 min led to an increase in artery tension to 2.2 ± 0.2 mN·mm^−1^ (*N* = 5) and 3.4 ± 0.4 mN·mm^−1^ (*N* = 5), respectively. Washout with PSS resulted in immediate onset of relaxation. In addition to the comparable levels of contraction achieved, the time course of blue light‐induced and KCl‐induced contractions was very similar, and each followed a double exponential time course, with a fast (*τ*_*f*_) and slow (*τ*_*s*_) component. These components were quantifiable during the long (7 min) stimulations. KCl stimulation was associated with *τ*_*f*_ of 7.7 ± 1.4 s (*N* = 5) and a *τ*_*s*_ of 122 ± 16 s (*N* = 5), whereas light stimulation was characterized by *τ*_*f*_ of 5.0 ± 0.7 s (*N* = 5) and *τ*_*s*_ of 117 ± 19 s (*N* = 5). These experiments showed that in agreement with the well‐established contractile effect of elevated [KCl]_e_, blue light stimulation also needed to be continuously maintained in order to sustain aortic smooth muscle contraction.

### Effect of ChR2(H134R) activation on the *V*_*m*_ of isolated VSMCs

**Figure 3:**
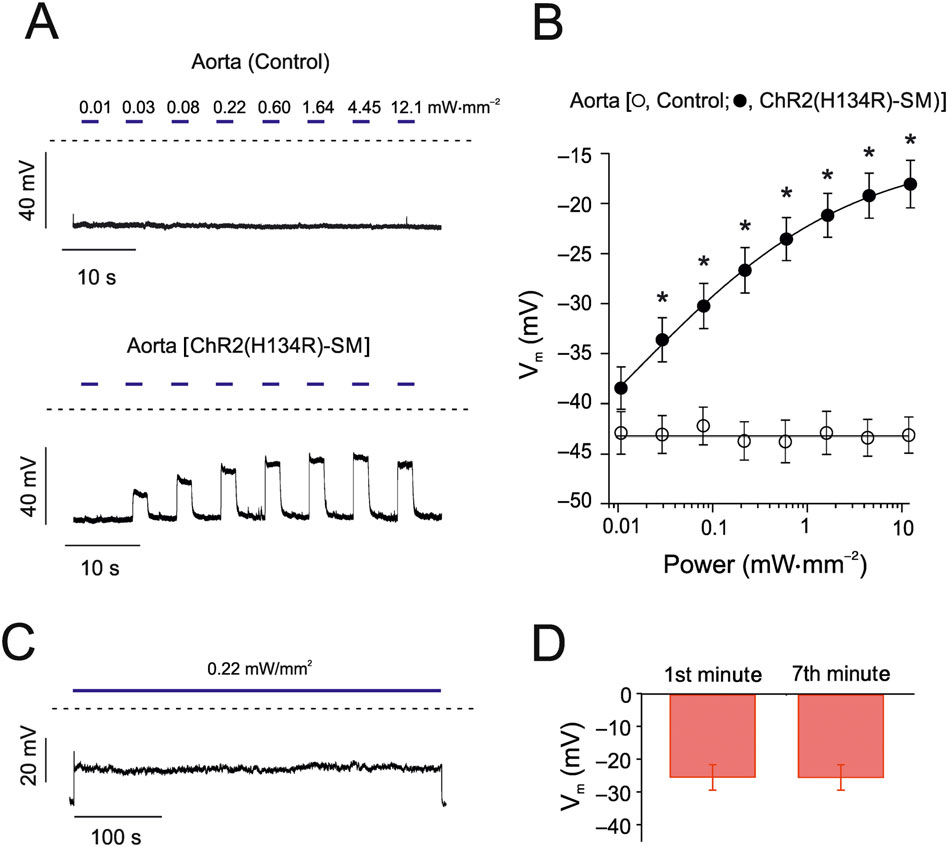
Effects of blue light on *V*_*m*_ in isolated aortic VSMCs. (A) *V*_*m*_ recordings of aortic VSMCs isolated from control and ChR2(H134R)‐SM mice in response to blue light stimulations (2 s) of various intensities, as indicated by the horizontal blue bars. The dashed lines indicate the zero voltage level. (B) Mean *V*_*m*_ versus blue light intensity relationships for aortic VSMCs isolated from control (*N* = 5, *n* = 12) or ChR2(H134R)‐SM mice (*N* = 11, *n* = 17). The curve through open circles (control) was drawn by the eye. The curve through filled circles (ChR2(H134R)‐SM) represents the best fit of the data with Equation (2). (C) *V*_*m*_ recording of an aortic VSMC isolated from ChR2(H134R)‐SM mouse in response to 7 min blue light stimulation (0.22 mW·mm^−2^). (D) Mean *V*_*m*_ measured during the first and seventh minute of exposure to blue light (0.22 mW·mm^−2^) (*N* = 6, *n* = 15). ^*^ *P* < 0.05, significantly different from control; Student's *t*‐test.

As outlined in the previous section, changes in arterial tone are expected to be linked to changes in *V*_*m*_ (Nelson *et al*., 1990). To quantitatively define the effects of ChR2(H134R) on the *V*_*m*_ of aortic VSMC, the perforated‐patch configuration in current‐clamp mode was used. The resting *V*_*m*_ of ChR2(H134R)‐SM aortic VSMCs was −40.2 ± 1.6 mV (*N* = 13), which was not significantly different from the resting *V*_*m*_ of VSMCs obtained from control mice (−42.2 ± 2.2 mV, *N* = 5) (Figure 3A, B). In response to light stimulations of up to 12.1 mW·mm^−2^, the membrane depolarized to −21.5 ± 1.3 mV (*N* = 11) in aortic VSMCs isolated from ChR2(H134R)‐SM mice (Figure 3). Cessation of light stimulation resulted in a prompt return to resting *V*_*m*_ (Figure 3A). The fit of the *V*_*m*_ versus light intensity relationship with Equation (2) yielded an EL_50_ of 0.2 ± 0.1 mW·mm^−2^ (*N* = 11), a slope factor (*v*) of 0.6 ± 0.1 (*N* = 11) and the maximal asymptotic *V*_*m*_ (*V*_max_) of −16.2 ± 2.6 mV (*N* = 11). In contrast, the *V*_*m*_ of aortic VSMCs obtained from control mice was not significantly affected by blue light (Figure 3A, B). Thus, the *V*_*m*_ of isolated aortic VSMCs expressing ChR2(H134R) can be optically clamped by exposure to blue light.

As outlined above, ChR2(H134R)‐mediated contraction of isolated aortic rings required continuous, prolonged exposures to blue light. Thus, the suitability of ChR2(H134R) to maintain *V*_*m*_ depolarization for prolonged periods of time (7 min) was also tested. The *V*_*m*_ of isolated aortic VSMCs in response to 0.22 mW·mm^−2^, an intensity that produced half‐maximal depolarization, remained clamped to approximately −27 mV during the 7 min illumination period (Figure 3C). This effect was quantified by measuring the *V*_*m*_ in the first and seventh minute of blue light exposure: no significant change in *V*_*m*_ was detected (Figure 3D).

*V*_*m*_ depolarization is expected to lead to activation of Ca_v_ channels, increased Ca^2+^ influx and VSMC contraction (Knot *et al*., 1996; Gollasch and Nelson, 1997; Jackson, 2000). To define the precise relationship between ChR2(H134R)‐induced depolarization and extent of Ca_v_ channel activation in our model, the voltage dependence of Ca_v_ channel activation and inactivation was determined in aortic VSMCs isolated from ChR2(H134R)‐SM mice (Supporting Information Figure S2). The steady‐state activation curve was characterized by a half‐activation potential (*V*_0.5,act_ *)* of 0.1 ± 2.0 mV (*N* = 8) and a slope factor (*s*_*a*_) of 8.9 ± 0.6 mV (*N* = 8). The steady‐state inactivation curve was characterized by a half‐inactivation potential (*V*_0.5,inact_) of −34.1 ± 1.4 mV (*N* = 7) and a slope factor (*s*_*i*_) of 11.5 ± 1.1 mV (*n* = 7). Window currents, the range of *V*_*m*_ in which Ca_v_ channels are active, spanned between approximately −40 and approximately +10 mV (Supporting Information Figure S2B). Thus, *V*_*m*_ depolarization due to ChR2(H134R) activation shifted the *V*_*m*_ from a resting value of approximately −40 mV, to values that fall within the window current range of Ca_v_ channels.

### Effects of light in the presence of phenylephrine

**Figure 4:**
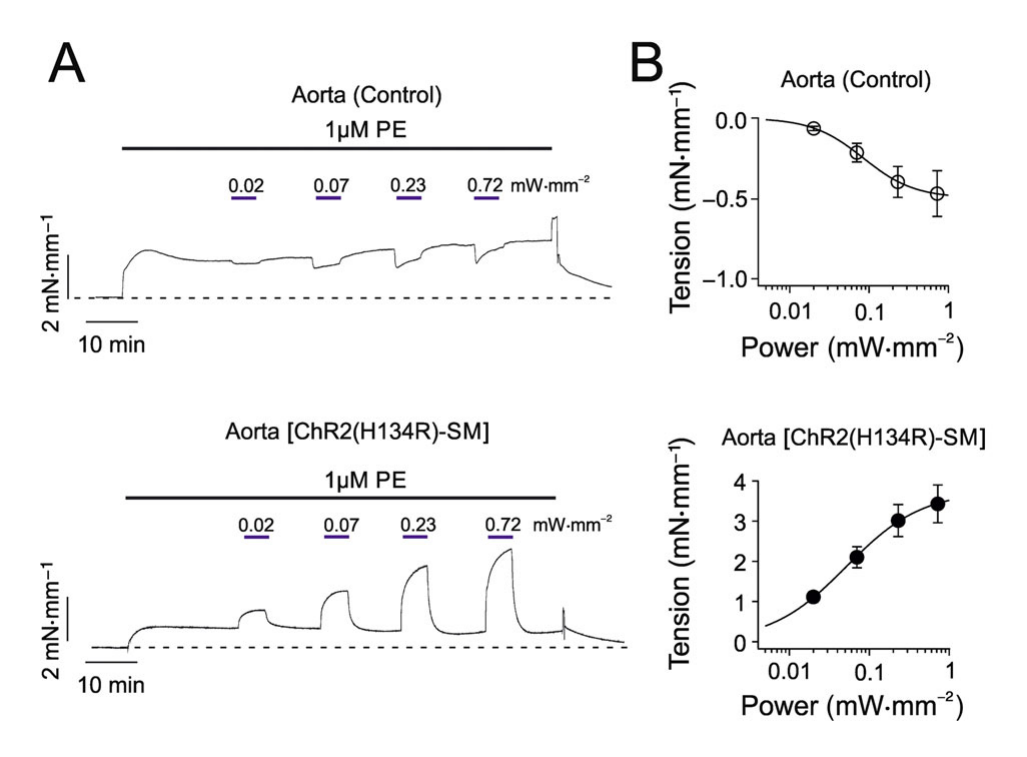
Effect of blue light on tension of isolated aortic rings in the presence of phenylephrine. (A) Isometric tension recordings obtained from aortic rings isolated from control or ChR2(H134R)‐SM mice, as indicated. Artery rings were exposed to phenylephrine (PE; 1 μM) and blue light as indicated by the black and blue horizontal bars respectively. The dashed lines indicate the basal tension level. (B) Mean tension versus blue light intensity relationships for aortic rings obtained from control (*N* = 5, *n* = 9) or ChR2(H134R)‐SM mice (*N* = 5, *n* = 10), as indicated. The smooth curves through symbols represent the best fit of the data with Equation (5).

The isolated artery ring experiments described above were performed in the absence of contractile agonists, such as phenylephrine . As outlined in the Introduction, in the presence of contractile agents, isolated wild‐type arterial smooth muscle has been reported to relax in response to blue light (Furchgott *et al*., 1955; Ehrreich and Furchgott, 1968; Sikka *et al*., 2014). The next set of experiments aimed to test whether ChR2(H134R)‐mediated depolarization and contraction could overcome the relaxing response of arteries to blue light. We began by quantifying the relaxation of aortic rings obtained from control mice in response to blue light. In the presence of phenylephrine (1 μM), aortic rings relaxed in a blue light intensity‐dependent manner (Figure 4A). For example, blue light intensity of 0.72 mW·mm^−2^ resulted in a ~0.5 mN·mm^−1^ reduction in artery ring tension (Figure 4B). Fit of the tension versus light intensity relationship with Equation (5) yielded an EL_50_ of 0.1 ± 0.1 mW·mm^−2^ (*N* = 5), a slope factor (*s*_*n*_) of 1.5 ± 0.1 (*N* = 5) and a maximal reduction in vessel tension (*T*_*ss*_) of −0.5 ± 0.2 (*N* = 5).

To establish whether opening of ChR2(H134R) was sufficient to counteract the relaxation described above, aortic rings isolated from ChR2(H134R)‐SM mice were exposed to blue light of increasing intensities in the presence of phenylephrine (1 μM). Under these conditions, aortic rings contracted in response to blue light (Figure 4A). For example, blue light of 0.72 mW·mm^−2^ elicited a ~3.5 mN·mm^−1^ increase in tension (Figure 4B). The fit of the tension versus blue light intensity relationship with Equation (5) yielded an EL_50_ of 0.1 ± 0.1 mW·mm^−2^ (*N* = 5), a slope factor (*s*_*n*_) of 0.9 ± 0.1 (*N* = 5) and a *T*_*ss*_ of 3.8 ± 0.6 mN·mm^−1^ (*N* = 5).

### Ionic mechanisms of ChR2(H134R)‐induced artery contraction

**Figure 5:**
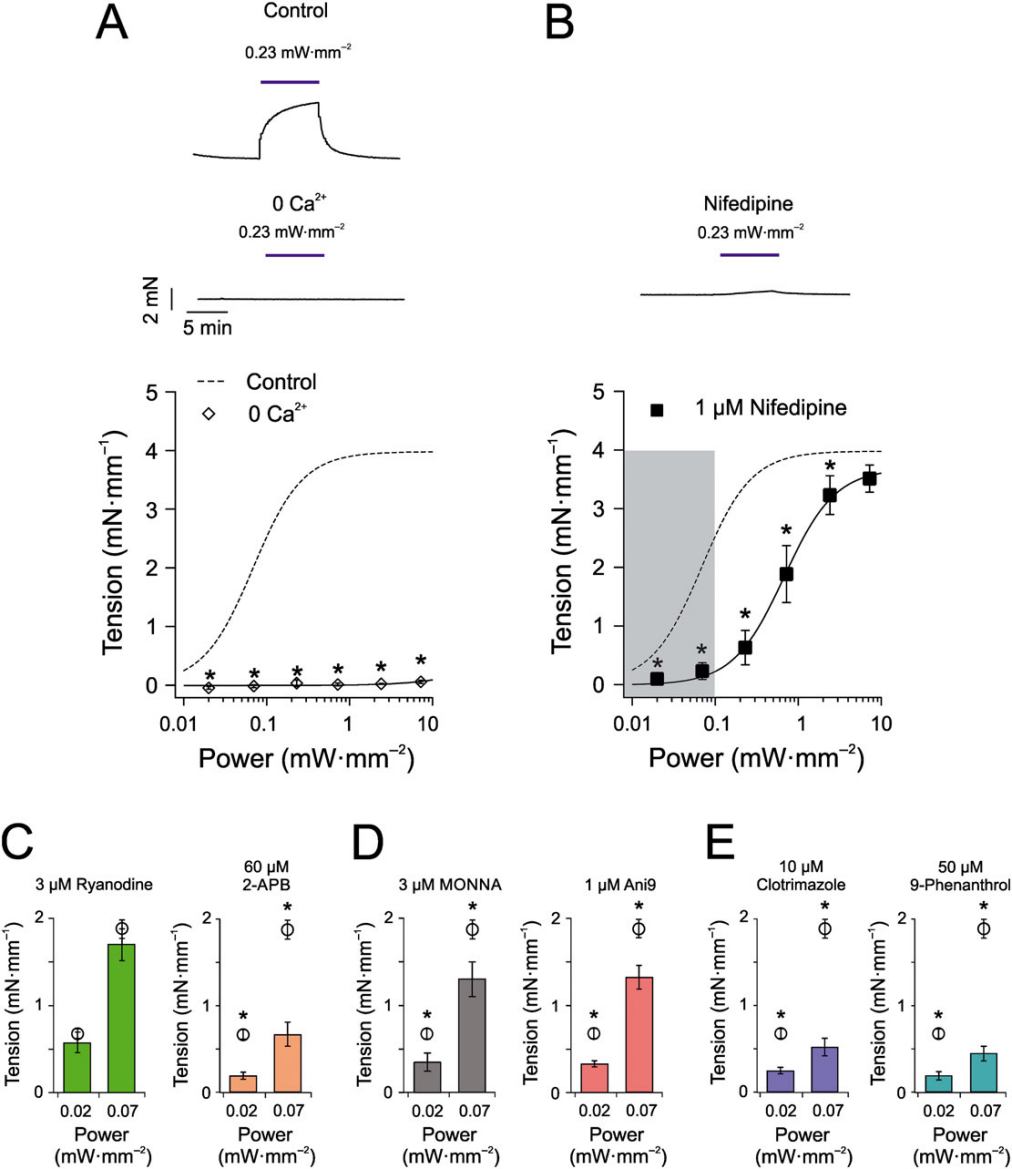
Effect of pharmacological ion channel modulation and ionic substitution on blue light‐induced tension of aortic rings obtained from ChR2(H134R)‐SM mice. (A) Top panels represent isometric tension recordings obtained from aortic rings isolated from ChR2(H134R)‐SM mice in response to 0.23 mW·mm^−2^ blue light in the presence of PSS (control) or a nominally Ca^2+^‐free PSS (0 Ca^2+^). The lower panel represents mean tension versus blue light intensity relationship for experiments conducted in Ca^2+^‐free PSS (*N* = 5, *n* = 10). The sigmoidal curve (dashed) is the Hill fit (Equation (5)) of the aortic ring tension versus blue light intensity relationship presented in Figure 2B. (B) Top panel represents isometric tension recordings obtained from an aortic ring isolated from ChR2(H134R)‐SM mice in response to 0.23 mW·mm^−2^ blue light in the presence of PSS supplemented with nifedipine (1 μM). The lower panel represents mean tension versus blue light intensity relationship for experiments conducted in PSS supplemented with nifedipine (1 μM) (*N* = 7, *n* = 8). Continuous sigmoidal curve through squares symbols indicates the best fit of the data with Equation (5). Dashed sigmoidal curve is the Hill fit (Equation (5)) of the aortic ring tension versus blue light intensity relationship presented in Figure 2B. (C) Mean tension in response to blue light stimulations in PSS supplemented with 3 μM ryanodine (*N* = 6, *n* = 8) or 60 μM 2‐APB (*N* = 6, *n* = 8), as indicated. Open circles refer to experiments conducted in control PSS solution and are replotted from Figure 2B. (D) Mean tension in response to blue light stimulations in PSS supplemented with either MONNA (3 μM) (*N* = 5, *n* = 10) or Ani9 (1 μM) (*N* = 5, *n* = 10). Open circles refer to experiments conducted in control PSS solution and are replotted from Figure 2B. (E) Mean tension in response to blue light stimulations in PSS supplemented with either clotrimazole (10 μM) (*N* = 5, *n* = 9) or 9‐phenanthrol (50 μM) (*N* = 5, *n* = 5). Open circles refer to experiments conducted in control PSS solution and are replotted from Figure 2B. ^*^ *P* < 0.05, significantly different from control; Student's *t*‐test.

The next series of myography experiments were conducted to characterize the ionic mechanism(s) underlying the ChR2(H134R)‐mediated contraction of aortic rings. To determine whether blue light‐induced contraction required influx of Ca^2+^ from the extracellular fluid, aortic rings obtained from ChR2(H134R)‐SM mice were exposed to blue light in Ca^2+^‐free PSS. Under these conditions, blue light stimulation produced a very small contraction. For example, the tension measured during 7.21 mW·mm^−2^ blue light stimulation was 0.1 ± 0.1 mN·mm^−1^ (*N* = 5) compared with 4.0 ± 0.1 mM·mm^−1^ (*N* = 32) in the presence of extracellular Ca^2+^ (Figure 5A).

The contraction of isolated aortic rings was dramatically reduced by application of the Ca_v_ channel blocker nifedipine at a concentration (1 μM) that blocks L‐type Ca^2+^ channels fully (Figure 5B) (Brayfield, 2014). Nifedipine shifted the EL_50_ of the tension versus light intensity relationship from ~0.1 to ~0.8 mW·mm^−2^, and the slope factor (*s*) slightly increased from *~*1.5 to ~2, while the maximal tension achieved (*T*_*ss*_) remained unaltered (~3.6 mN·mm^−1^) (Table 3). Note that nifedipine (1 μM) almost completely abolished the contraction of aortic rings in response to elevated [KCl]_e_; 70 mM [KCl]_e_ induced contraction of 0.2 ± 0.1 mN·mm^−1^ in the presence of nifedipine (not shown) compared with ~4.0 mN·mm^−1^ in the absence (Supporting Information Figure S1).

**Table 3:** Parameters for the tension versus blue light relationships for aortic rings obtained from ChR2(H134R)‐SM mice in the presence of nifedipine

	ChR2(H134R)‐SM mice
	Aorta
EL_50_ (mW·mm^−2^)	0.8 ± 0.2 (*N* = 7, *n* = 8)*
*s*	2.0 ± 0.3 (*N* = 7, *n* = 8)*
*T*_*ss*_ (mN·mm^−1^)	3.6 ± 0.3 (*N* = 7, *n* = 8)

Parameters (EL_50_, *s* and *T*_max_) obtained from the Hill fit (Equation (5)) of the tension versus blue light intensity relationship for aortic rings obtained from ChR2(H134R)‐SM mice.

* *P* < 0.05,significantly different from data without nifedipine, shown in Table 2; Student's *t*‐test.

Nifedipine is a light‐sensitive compound that photodegrades into a 2‐nitroso derivative when exposed to daylight (Brayfield, 2014). Therefore, the possibility that nifedipine was degraded during exposure to blue light was examined *via* a separate set of functional electrophysiological experiments. The extracellular whole‐cell patch‐clamp solution containing nifedipine (1 μM) was illuminated with high‐intensity blue light (2.4 mW·mm^−2^) for 10 min. The extent to which Ca_v_ current in isolated aortic VSMCs was inhibited by this solution was indistinguishable from that caused by a nifedipine‐containing solution that was not exposed to light (Supporting Information Figure S2C). Thus, exposure of nifedipine to blue light did not alter its inhibitory effect on Ca_V_ channels in these experiments.

The isometric tension recordings described above demonstrated that in response to light stimulations below ~0.1 mW·mm^−2^, aortic ring contraction required Ca_v_ channel activation. Presumably contractions triggered by blue light stimulations of greater power would involve, at least in part, Ca^2+^ influx through ChR2(H134R) and would result in a level of contraction, which may not be reached under normal physiological conditions. To evaluate the maximal degree of tension that the artery may develop *in vivo*, we examined the response of isolated aortic artery rings to the physiological agonist noradrenaline. Supporting Information Figure S3 shows that the maximal tension triggered by this agonist was ~2.4 mN·mm^−1^. This corresponds to the contraction mediated by ChR2(H134R) in response to blue light of 0.1 mW·mm^−2^. Thus, we considered light stimulations <0.1 mW·mm^−2^ those that elicit a contraction that resemble, in terms of magnitude and underlying Ca^2+^ handling mechanisms, physiological contractions.

To gain further understanding of the ionic mechanisms underlying ChR2(H134R)‐mediated contraction within the identified blue light stimulation range (i.e. <0.1 mW·mm^−2^), we examined the light response in the presence of a series of pharmacological modulators. To determine the contribution of http://www.guidetopharmacology.org/GRAC/FamilyDisplayForward?familyId=125 (RyR)‐mediated Ca^2+^‐induced Ca^2+^ release (CICR) to the ChR2(H134R)‐induced contraction of aortic rings, http://www.guidetopharmacology.org/GRAC/LigandDisplayForward?ligandId=4303 (3 μM) was added to the PSS solution 25 min prior to blue light stimulation to exhaust ryanodine‐sensitive Ca^2+^ stores (Ito *et al*., 1986). Figure 5C illustrates that this intervention did not significantly affect the arterial response to blue light. The contribution of http://www.guidetopharmacology.org/GRAC/LigandDisplayForward?ligandId=4222 to CICR was also tested by incubating vessels in http://www.guidetopharmacology.org/GRAC/LigandDisplayForward?ligandId=2433) (60 μM). Figure 5C shows that inhibition of IP_3_ receptors with 2‐APB significantly reduced the arterial response to blue light. Thus, Ca_v_‐mediated Ca^2+^ entry and IP_3_‐mediated CICR are likely to be the major sources of Ca^2+^ leading to arterial contraction in response to blue light of <0.1 mW·mm^−2^ in ChR2(H134R)‐SM aortic rings.

The CaCC coded by the *Tmem16a* gene represents a key depolarizing mechanism in vascular smooth muscle (Manoury *et al*., 2010; Bulley and Jaggar, 2014; Hübner *et al*., 2015; Matchkov *et al*., 2015). CaCCs in VSMCs are typically activated by IP_3_‐mediated Ca^2+^ release (Manoury *et al*., 2010; Bulley and Jaggar, 2014; Hübner *et al*., 2015; Matchkov *et al*., 2015). To investigate whether TMEM16A channels participate in the blue light‐induced contraction in arteries obtained from ChR2(H134R)‐SM mice, the TMEM16A inhibitors MONNA (3 μM) (Oh *et al*., 2013) or Ani9 (1 μM) (Seo *et al*., 2016) were added to the PSS. Pharmacological blockade of TMEM16A channels with these agents significantly reduced the contractile response to blue light stimulation by ~0.5‐fold and ~0.3‐fold at 0.02 and 0.07 mW·mm^−2^ respectively (Figure 5D). Another Ca^2+^‐activated depolarizing channel types in VSMCs are the TRPM channels (Guibert *et al*., 2011). Their involvement was tested by inhibiting them using the TRPM blocker http://www.guidetopharmacology.org/GRAC/LigandDisplayForward?ligandId=2330 (10 μM) (Zholos, 2010). This caused significant reduction of blue light‐induced contraction by up to ~0.7‐fold (Figure 5E). The less selective channel blocker http://www.guidetopharmacology.org/GRAC/LigandDisplayForward?ligandId=4114 (50 μM), which blocks TRPM and TMEM16A channels caused up to ~0.8‐fold reduction of contraction (Figure 5E).

### Blue light evoked changes in intracellular pH

**Figure 6:**
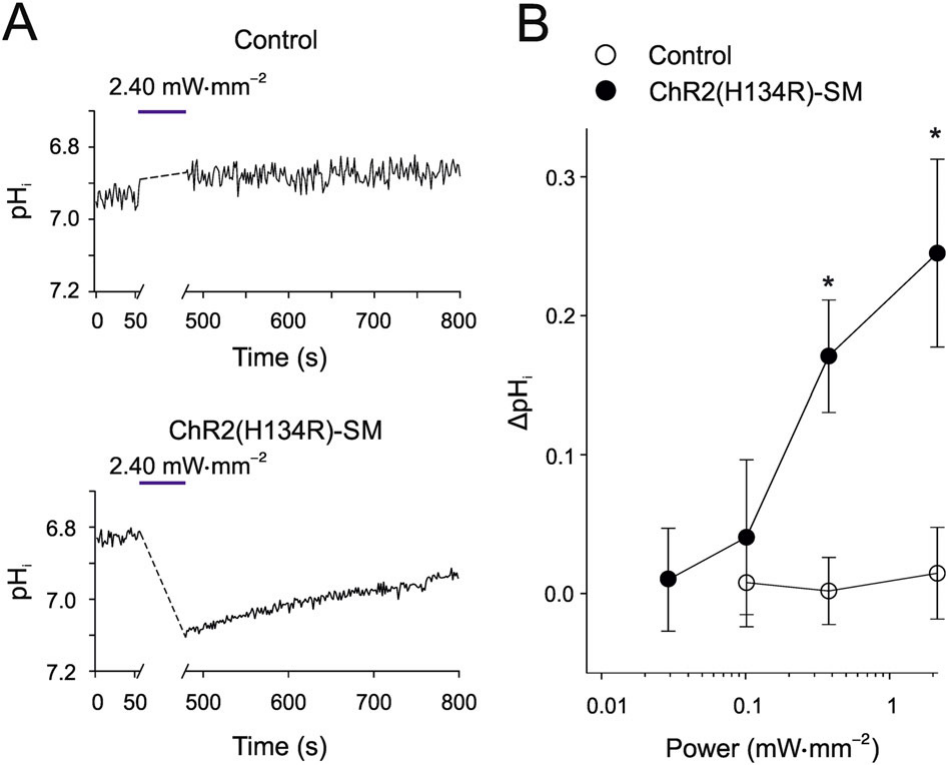
Effect of blue light on pH_i_ of aortic VSMCs. (A) pH_i_ measurements in individual aortic VSMCs, isolated from control and ChR2(H134R)‐SM mice, before and after a 7 min exposure to blue light of different intensities, as indicated. The horizontal blue bars in each panel indicate the 7 min illumination period. (B) Mean blue light‐induced pH_i_ change versus blue light intensity relationship for VSMCs isolated from ChR2(H134R)‐SM mice (*N* = 8, *n* = 201) and control mice (*N* = 5, *n* = 215). ^*^ *P* < 0.05, significantly different from control; Student's *t*‐test.

As ChR2(H134R) is a ion channel highly permeable to H^+^, prolonged activation may lead to a net transmembrane H^+^ flux and changes in pH_i_. To test this, pH_i_ was measured in isolated aortic VSMCs obtained from ChR2(H134R)‐SM and control mice before and after 7 min light stimulation of various intensities (Figure 6). The pH_i_ prior to light stimulation was 6.9 ± 0.1 (*N* = 5) in cells isolated from ChR2(H134R)‐SM mice. In VSMCs isolated from control mice, the starting pH_i_ was 6.8 ± 0.1 (*N* = 5). Figure 6 shows that no significant change in pH_i_ was observed at low‐intensity blue light (<0.1 mW·mm^−2^). At higher intensities, however, the light protocol evoked significant alkalinization of the cytoplasm, by up to ~0.25 pH units (Figure 6B).

### Comparison of light‐induced and agonist‐induced contraction

**Figure 7:**
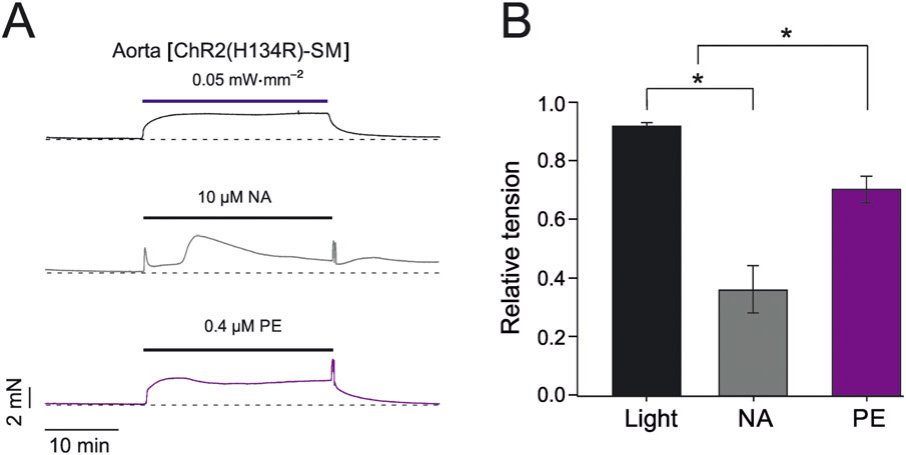
Comparison of blue light‐induced and agonist‐induced contraction of aortic rings obtained from ChR2(H134R)‐SM mice. (A) Representative isometric tension recordings obtained from aortic rings isolated from ChR2(H134R)‐SM mice. Aortic rings were exposed to blue light, phenylephrine (PE) or noradrenaline (NA) for 25 min, as indicated by the horizontal bars. (B) Mean stability of contraction expressed as the ratio between the maximal and minimal level of contraction elicited by blue light (*N* = 5, *n* = 8), PE (*N* = 5, *n* = 11) or NA (*N* = 5, *n* = 11). ^*^ *P* < 0.05, significantly different as indicated; one‐way ANOVA.

The previous sets of experiments identified the range of blue light intensities within which ChR2(H134R) activation resulted in VSMCs depolarization, Ca_v_ and TMEM16A channel activation and arterial contraction in the absence of pronounced changes in pH_i_ or Ca^2+^ entry through ChR2(H134R). We examined the contractile response of isolated aortic rings to prolonged (25 min) stimulation with a light intensity (0.05 mW·mm^−2^), which falls within this range. The response was then compared with those elicited by contractile agonists such as phenylephrine (0.4 μM) and noradrenaline (10 μM). These agonist concentrations were selected as they evoked an average contraction of 1.4 ± 0.1 mN·mm^−1^ (noradrenaline, *N* = 5) and 1.1 ± 0.1 mN·mm^−1^ (phenylephrine, *N* = 5), which were comparable with a tension of 1.2 ± 0.1 mN·mm^−1^ (*N* = 5) induced by 0.05 mW·mm^−2^ blue light. Blue light stimulation triggered a contraction that rapidly reached a maximal, stable steady‐state value. In contrast, noradrenaline produced a transient contraction followed by fluctuating levels of tension (Figure 7A). Phenylephrine ‐induced contraction was characterized by a peak followed by a slow drop and then subsequent rise in tension. The nadir to peak ratio measured in each artery ring was used as a measure of the stability of the tension induced by the various stimuli. This ratio was 0.9 ± 0.1 (*N* = 5), 0.4 ± 0.1 (*N* = 5) and 0.7 ± 0.1 (*N* = 5) for blue light, noradrenaline and phenylephrine respectively (Figure 7B). Thus, blue light stimulation produced a more stable response than those to the contractile agonists.

## Discussion

This study provides a comprehensive analysis of the blue light sensitivity of isolated VSMCs and artery rings obtained from ChR2(H134R)‐SM mice. Activation of the ChR2(H134R) channels caused *V*_*m*_ depolarization, activation of Ca_v_, TMEM16A and TRPM channels. In intact artery rings, this resulted in an increase in artery tone. In the presence of blue light of intensities <0.1 mW·mm^−2^, the artery ring contraction was completely prevented by nifedipine. This indicated that Ca_v_ channel activation was mandatory for contraction in response to blue light intensities. Within this light intensity range, TMEM16A and TRPM channels and IP_3_ receptors contributed to the contractile response. Furthermore, the pH_i_ remained stable. In contrast, light stimuli greater than 0.1 mW·mm^−2^ led to contractions, even in the presence of Ca_v_ channel blockade, suggesting that significant Ca^2+^ influx through ChR2(H134R) channels might be occurring. In addition, the pH_i_ of isolated aortic VSMCs was significantly alkalinized. Thus, this study determined the range of light intensities (<0.1 mW·mm^−2^) that enabled modulation of the *V*_*m*_ and artery tone of ChR2(H134R)‐SM mice primarily *via* recruitment of endogenous channels while minimizing any disturbances to pH_i_. This experimental analysis demonstrates the ChR2(H134R)‐SM mice as a powerful experimental model to selectively control the excitability of VSMCs.

### ChR2(H134R) expression and current amplitude in isolated VSMCs

The level of ChR2(H134R) expression in VSMCs decreased in the order pulmonary artery ≈ aorta > mesenteric artery. The amplitude of the current density also followed this order. Possible causes of different ChR2(H134R) protein expression in different VSMC types include (i) variable levels of transcription factors acting on the transgelin promoter, which in turn controls the expression of the Cre recombinase and/or (ii) differences in how proteins are trafficked and expressed in different VSMCs types. Regardless of the underlying causes of variable expression of ChR2(H134R) in different artery types, the degree of expression in the arteries tested was sufficient to allow maximal or near‐maximal contraction in all artery types tested.

The time course of the change in tension of aortic rings in response to blue light or elevated [KCl]_e_ was remarkably similar. Furthermore, continuous application of either stimulus was necessary for the tension of isolated aortic rings to reach a maximal, steady‐state value. These observations suggest that activated ChR2(H134R) channels acted as a depolarizing mechanism (providing an ‘optical voltage‐clamp’ method) in a similar way to high [KCl]_e_ (constituting ‘chemical voltage clamp’). Vascular optogenetics provides significant advantages compared with depolarization achieved by elevation of [KCl]_e_. For instance, in experiments involving whole organs, elevated KCl could not be applied selectively to VSMCs without affecting neighbouring cells and tissues. In contrast, optogenetics can enable selective control of VSMC excitability. Furthermore, elevation of extracellular K^+^ concentration may affect transport mechanisms such as the Na^+^/K^+^ ATPase, which could lead to an alteration in the intracellular Na^+^ concentration. In turn, this may affect other secondary transport mechanisms as well as the action of numerous enzymes and intracellular proteins (Page and Di Cera, 2006).

### ChR2(H134R)‐induced and agonist‐induced contraction of isolated artery rings

Earlier studies have indicated that systemic arteries obtained from wild‐type animals pre‐constricted with noradrenaline or phenylephrine, relaxed in response to blue light (Furchgott *et al*., 1955; Ehrreich and Furchgott, 1968; Sikka *et al*., 2014). A key mechanism involved in this response was the activation of http://www.guidetopharmacology.org/GRAC/ObjectDisplayForward?objectId=2758 receptors (Sikka *et al*., 2014). Dilation in response to blue light was also observed in murine cerebral arterioles although the molecular mechanism and signalling pathway involved in photodilation in this circulation remains unclear (Rungta *et al*., 2017). We have determined that ChR2(H134R)‐mediated contraction overcame the native relaxation in murine systemic arteries in response to blue light. This is a prerequisite for the use of vascular optogenetic *in vivo* as the arteries are exposed to varying levels of circulating or locally released catecholamines, depending on the extent of sympathetic stimulation. This information indicates a possible use of vascular optogenetics *in vivo* as a tool to control vessel tone.

Isolated aortic artery rings obtained from ChR2(H134R)‐SM mice responded to blue light with a stable contraction. In contrast, fluctuations of isolated artery tone were observed in response to receptor agonists such as phenylephrine or noradrenaline. This may have significant implications for pharmacology and drug discovery. The potency of new vasodilating compounds might be more difficult to assess in vessels pre‐constricted with agents that elicit a changing level of vessel tone. This is because spontaneous variation in tone may complicate the quantification of the effects of vasodilating drugs. The stability of the blue light‐induced contraction could therefore aid the assessment of new vasodilating drugs on isolated arteries.

### Investigating the ionic mechanisms underlying ChR2(H134R)‐induced artery contraction

Ca_v_ channels are a principal and well‐established route of Ca^2+^ influx in VSMCs (Jackson, 2000; Cribbs, 2001). In isolated aortic VSMCs, Ca_v_ channel window currents spanned from approximately −40 to +10 mV while the resting *V*_*m*_ was approximately −40 mV. This indicated that a small blue light‐dependent depolarization from the resting *V*_*m*_ was sufficient to bring the *V*_*m*_ into a range where Ca_v_ channels were active. Therefore, the ChR2(H134R) channels served, primarily, as a switch for Ca_v_ channel activation. CICR mediated by IP_3_ receptors but not RyR contributed to the blue light‐induced artery contraction. This suggests that the ChR2(H134R) channels are not directly juxtaposed to RyRs in the endoplasmic reticulum.

It is likely that 2‐APB inhibits targets other than IP_3_R. Specifically, 2‐APB may also inhibit TRP channels, including http://www.guidetopharmacology.org/GRAC/FamilyDisplayForward?familyId=78 channels (Xu *et al*., 2005). These channels however are activated by PIP_2_ breakdown, which usually takes place in response to G_q_PCR stimulation (Gonzalez‐Cobos and Trebak, 2010). Thus, TRPC channels may not be fully active during the ChR2(H134R)‐induced contraction and 2‐APB may not be eliciting its actions *via* blockade of TRPC channels. Another off‐target effect of 2‐APB is the inhibition of gap junctions (Bai *et al*., 2006). One of the most abundantly expressed connexins in smooth muscle is http://www.guidetopharmacology.org/GRAC/FamilyDisplayForward?familyId=121#728 (Figueroa and Duling, 2009), which has relatively low sensitivity to 2‐APB (IC_50_ = 52 μM)(Bai *et al*., 2006). Furthermore, gap junctions are involved in the electrical propagation among neighbouring cells, but blue light‐induced depolarization will affect all individual VSMCs. Under these conditions, net transmission across gap junctions is unlikely to substantially affect contraction.

Application of the Ca_v_ channel blocker, nifedipine, shifted the tension versus blue light intensity response curve to the right by an order of magnitude. At light intensities <0.1 mW·mm^−2^, nifedipine completely prevented the blue light‐induced contraction of aortic rings isolated from ChR2(H134R)‐SM mice. However, at higher light intensities, a significant Ca^2+^ current may be directly mediated by ChR2(H134R) channels and thus triggering contraction even in the presence of nifedipine. This non‐physiological Ca^2+^ entry could potentially lead to Ca^2+^ overload, which could cause functional and histological alterations in the vessel wall (Atkinson, 1992; Fleckenstein‐Grün *et al*., 1992).

The TMEM16A channel blockers Ani9 and MONNA reduced the blue light‐induced contraction of isolated aortic rings. This may suggest that Ca^2+^ entry through Ca_v_ or ChR2(H134R) channels, in turn, activated TMEM16A channels. TMEM16A channels act as depolarizing channels in VSMCs due to the raised intracellular Cl^−^ concentration ([Cl^−^]_i_), which is maintained at high levels by active transport mechanisms. This raises the equilibrium potential for Cl^−^ above the resting *V*_*m*_ of the cell (Manoury *et al*., 2010; Bulley and Jaggar, 2014; Hübner *et al*., 2015; Matchkov *et al*., 2015). Thus, TMEM16A opening may also contribute to the blue light‐induced depolarization of VSMCs and arterial contraction. Our pharmacological analysis using clotrimazole indicated that Ca^2+^‐activated TRPM channels also contributed to the blue light‐induced contraction.

Clotrimazole may also block intermediate conductance K^+^ (IK_Ca_) channels (Wulff *et al*., 2001). These channels are especially expressed in endothelial cells (ECs), where they contribute to NO generation and endothelium‐dependent hyperpolarization (Félétou, 2009). Thus, their inhibition would presumably enhance contraction and antagonize the reduction in blue light‐induced contraction caused by this agent. Clotrimazole has also been shown to partially (20%) block cardiac Ca_v_ channels (Thomas *et al*., 1999). This may contribute to a small fraction of the effect of clotrimazole on light‐induced contraction in aortic rings. The TRPM4 blocker 9‐phenanthrol reduced the extent of contraction to a similar degree to clotrimazole. The pharmacology of TRPM channels is currently limited in terms of specificity, and 9‐phenanthrol has recently been shown to also block TMEM16A channels (Burris *et al*., 2015). However, the block seen with 9‐phenanthrol is significantly greater than that observed with either MONNA or Ani9 suggesting a clear role for TRPM4 channels in blue light‐induced contraction.

### Blue light‐driven intracellular alkalinization

The ChR2(H134R) channels are highly permeable to H^+^ (Nagel *et al*., 2003; Lin *et al*., 2009; Berndt *et al*., 2010, 2011; Gradmann *et al*., 2011). We demonstrated that prolonged activation of ChR2(H134R) channels in response to high‐intensity (>0.1 mW·mm^−2^) blue light stimulations produced intracellular alkalinization. These changes in pH_i_ are consistent with the fact that the average equilibrium potential for H^+^ (E_H+_) in the aortic VSMCs was approximately −29 mV under our experimental conditions, based on the Nernst equation for intracellular and extracellular pH of ~6.9 and 7.4 respectively. Thus, when *V*_*m*_ rises above −29 mV, as occurs in response to blue light intensities >0.1 mW·mm^−2^, outward H^+^ current through ChR2(H134R) channels is expected to raise pH_i_. We argue that a small depolarization of the *V*_*m*_ below approximately −29 mV, associated with blue light intensity lower than ~0.1 mW·mm^−2^, did not lead to significant acidification of pH_i_ as ChR2(H134R) channels were only partly activated (i.e. had a low open probability). Furthermore, abundantly expressed Na^+^/H^+^ exchanger activity in VSMCs (Aalkjaer and Peng, 1997) is likely to offset any H^+^ influx through ChR2(H134R) channels.

The effect of a change in pH_i_ by >0.1 pH units can substantially alter the sensitivity of VSMCs to humoral factors (Wray and Smith, 2004; Wakabayashi *et al*., 2006; Boedtkjer and Aalkjaer, 2012; Boedtkjer *et al*., 2016) as well as affect VSMC migration (Boedtkjer *et al*., 2016) and gene transcription (Wakabayashi *et al*., 2006). Thus, prolonged activation of ChR2(H134R) channels by high‐intensity blue light *in vivo* may lead to a pH‐dependent alteration in arterial histology and function.

### Implications for vascular pharmacology and therapeutic drug discovery

Vascular optogenetics has many potential applications including serving as an experimental tool to control blood perfusion to specific organs or tissues (Wu *et al*., 2015). Thus, organ ischaemia could be experimentally reproduced by inducing vessel constriction *in vivo* in experimental animals, potentially *via* the use of remotely controlled, wireless microLEDs implanted s.c. (Kim *et al*., 2013). This may lead to important implications for biomedical research by aiding the identification of new drugs or treatments that could counter ischaemia. As outlined above, the stable contraction obtained in isolated arteries may also facilitate drug testing *ex vivo*. At a single‐cell level, the possibility of controlling *V*_*m*_ optically could enable high‐throughput screening of drugs at various *V*_*m*_, including depolarized *V*_*m*_ typically associated with diseases of altered vessel tone (Platoshyn *et al*., 2000; Wellman *et al*., 2001). It can be envisaged that viral‐mediated ChR2(H134R) expression could be obtained in cultured human cells to be used in drug testing. This may help reducing, and to an extent replacing, the use of animals during drug screening studies. Our study demonstrates that vascular optogenetics is especially viable within a well‐defined range of blue light of low intensity within which activation of ChR2(H134R) channels serves merely as a switch for activation of other, endogenous, membrane transport mechanisms.

Vascular optogenetics has the potential to further our understanding of the physiological role of electrical coupling between different cell types (such as VSMCs and ECs) within the arterial wall. Investigating the electrical coupling between ECs and VSMCs in intact vessels has presented significant technical challenges. For instance, injection of current in ECs using sharp microelectrodes may result in non‐uniform control of *V*_*m*_. Voltage clamping of cell membrane is also restricted by the limited current‐passing ability of the microelectrodes. In addition, electrical stimulation may also initiate irreversible Faradaic reactions, potentially leading to the formation of gases such as H_2_, O_2_ or Cl_2_, which are likely to harm the cell (Merrill *et al*., 2005). Optogenetics may enable a non‐contact stimulation of specific cell types in the arterial wall for prolonged periods of time and without these limitations.

In 2015, a Cl^−^‐selective and H^+^‐impermeable ChR2 mutant was identified and termed iChloC (Wietek *et al*., 2015). Due to the high intracellular Cl^−^ concentration in VSMCs, activation of a light‐activated Cl^−^ channel is expected to evoke *V*_*m*_ depolarization, activation of Ca_v_ channels and smooth muscle contraction. Thus, we predict that iChloC could serve as a depolarizing mechanism without the changes in pH_i_ associated with the H^+^ permeability of ChR2(H134R) or the potential changes in intracellular Ca^2+^ due to the ChR2(H134R)‐mediated Ca^2+^ current. The use of iChloC may enable extension of vascular optogenetics to a much broader range of light stimulations and, consequently, enable a broader extent of artery tone regulation. There is no doubt that optogenetics has the potential to trigger new investigations in vascular research. It can be envisaged that opsins that cause *V*_*m*_ hyperpolarization might lead to vessel relaxation. These opsins might have the potential to be utilized in gene therapy in human subjects. This might have important implications for the treatment of widespread vascular diseases associated with altered vessel tone.

## Author contributions

P.T. initiated and directed the project and trained and supervised the team members throughout the project; P.S. trained N.J.G.R. in pH imaging; N.J.G.R., C.M.T., P.S. and P.T. designed the experiments. N.J.G.R., C.M.T. and H.G. conducted the experiments. N.J.G.R., C.M.T. and P.T. analysed the experiments. N.J.G.R. prepared all the figures. All authors discussed and interpreted the results. N.J.G.R. and P.T. wrote the manuscript, which was subsequently reviewed and approved by all the authors. P.T. obtained funding.

## Conflict of interest

The authors declare no conflicts of interest.

## Declaration of transparency and scientific rigour

This http://onlinelibrary.wiley.com/doi/10.1111/bph.13405/abstract acknowledges that this paper adheres to the principles for transparent reporting and scientific rigour of preclinical research recommended by funding agencies, publishers and other organisations engaged with supporting research.

## Supporting information


**Figure S1** Effects of [KCl]_e_ on the tension of rings of various artery types isolated from ChR2(H134R)‐SM mice. Mean tension versus [KCl]_e_ relationships for the different artery types (aortic, A, (*N*=20, *n*=32); mesenteric, B, (*N*=5, *n*=11) and pulmonary, C, (*N*=5, *n*=11)). The curves through the filled symbols represent the best fit of the data with Eq. 5.
**Figure S2** Ca_V_ channel currents in isolated aortic VSMCs A. Representative whole‐cell Ca_V_ channel currents obtained from aortic VSMCs isolated from ChR2(H134R)‐SM mice in response to the stimulation protocol shown in the top panel. For clarity, only the currents elicited every 20 mV are displayed. B. Mean steady‐state activation (*N*=8, *n*=15) and inactivation (*N*=7, *n*=12) curves for Ca_V_ channels measured in aortic VSMCs. Smooth curves represent the fit of the data using Eq. 3 and 4 (activation and inactivation, respectively). Grey bar highlights the range of *V*_*m*_ that intact VSMCs can experience as a result of ChR2(H134R)‐mediated depolarisations. C. Mean whole‐cell Ca_V_ currents versus *V*_*m*_ relationships in the absence (*N*=8, *n*=15) or presence of 1 μM nifedipine (Nif) which was either kept in the dark (*N*=5, *n*=9) or exposed to blue light (2.4 mW/mm2) for 10 min (light conditioned) (*N*=6, *n*=20), as indicated. Curves through symbols represent the best fit of the data with Eq. 3.
**Figure S3** Effects of noradrenaline on the tension of aortic rings isolated from ChR2(H134R)‐SM mice. Mean tension versus noradrenaline concentration ([NA]) relationship (*N*=5, *n*=8). The curve through the filled symbols represent the best fit of the data with Eq. 5.
**Table S1** Parameters for tension versus [KCl]_e_ relationships for artery rings obtained from ChR2(H134R)‐SM mice.
**Table S2** Parameters for tension versus [NA] relationships for artery rings obtained from ChR2(H134R)‐SM mice.
