# Supplementary material for: Defining the ionic mechanisms of optogenetic control of vascular tone by channelrhodopsin‐2
Source: Br J Pharmacol. 2018 Apr 17;175(11):2028–45. doi: 10.1111/bph.14183 (PMC5979753; doi:10.1111/bph.14183)
Supplement: Supplementary file 1 — Figure S1 Effects of [KCl]e on the tension of rings of various artery types isolated from ChR2(H134R)‐SM mice. Mean tension versus [KCl]e relationships for the different artery types (aortic, A, (N=20, n=32); mesenteric, B, (N=5, n=11) and pulmonary, C, (N=5, n=11)). The curves through the filled symbols represent the best fit of the data with Eq. 5. Figure S2 CaV channel currents in isolated aortic VSMCs A. Representative whole‐cell CaV channel currents obtained from aortic VSMCs isolated from ChR2(H134R)‐SM mice in response to the stimulation protocol shown in the top panel. For clarity, only the currents elicited every 20 mV are displayed. B. Mean steady‐state activation (N=8, n=15) and inactivation (N=7, n=12) curves for CaV channels measured in aortic VSMCs. Smooth curves represent the fit of the data using Eq. 3 and 4 (activation and inactivation, respectively). Grey bar highlights the range of V m that intact VSMCs can experience as a result of ChR2(H134R)‐mediated depolarisations. C. Mean whole‐cell CaV currents versus V m relationships in the absence (N=8, n=15) or presence of 1 μM nifedipine (Nif) which was either kept in the dark (N=5, n=9) or exposed to blue light (2.4 mW/mm2) for 10 min (light conditioned) (N=6, n=20), as indicated. Curves through symbols represent the best fit of the data with Eq. 3. Figure S3 Effects of noradrenaline on the tension of aortic rings isolated from ChR2(H134R)‐SM mice. Mean tension versus noradrenaline concentration ([NA]) relationship (N=5, n=8). The curve through the filled symbols represent the best fit of the data with Eq. 5. Table S1 Parameters for tension versus [KCl]e relationships for artery rings obtained from ChR2(H134R)‐SM mice. Table S2 Parameters for tension versus [NA] relationships for artery rings obtained from ChR2(H134R)‐SM mice. [file BPH-175-2028-s001.pdf]

N.J.G. Rorsman, C.M. Ta, H. Garnett, P. Swietach and P. Tammaro

SUPPLEMENTARY MATERIAL

ADDITIONS TO THE METHODS

Generation of transgenic mice

Transgenic mice had either: 1) eYFP-tagged ChR2(H134R) coding sequence (ChR2(H134R)-eYFP) inserted downstream of a loxP-flanked STOP cassette within the Gt(ROSA)26Sor locus (012569, Jackson Laboratories), or 2) the Cre recombinase coding sequence under the control of mouse transgelin (smooth muscle protein 22-α) promoter (004746, Jackson Laboratories). Homozygous mice from strain 1 and 2 were crossed. In the offspring, the removal of loxP-flanked STOP cassette by Cre recombinase led to selective expression of ChR2(H134R)-eYFP in SMCs. These mice were termed ChR2(H134R)-SM. Strain 1 mice were used as control. Adult mice (8-15 weeks) were culled by cervical dislocation in accordance with UK Home Office guidelines.

PCR for genotyping involved the following pairs of primers: 1) ACATGGTCCTGCTGGAGTTC (forward) and GGCATTAAAGCAGCGTATCC (reverse), to detect ChR2(H134R) and 2) GCGGTCTGGCAGTAAAAACTATC (forward) and GTGAAACAGCATTGCTGTCACTT (reverse), to detect Cre recombinase. Genomic DNA was extracted from ear biopsies using standard procedures outlined in (Sambrook and Russell, 2001). PCR reaction involved the following steps: 1) initialising step: 94°C, 5 min; 2) denaturation step: 94°C, 30 s; 3) annealing step: 61°C, 60 s; 4) extension step: 72°C, 60 s. Step 2-4 were repeated 38 times. The final extension time was 72°C for 7 min. The PCR products were visualised on a 1% agarose gel.

Artery dissection and cell isolation

Thoracic aorta and small (second and third order) mesenteric and pulmonary arteries were isolated and connective tissue removed in cold physiological salt solution (PSS). PSS contained (in mM): 122 NaCl, 5 KCl, 10 HEPES, 0.5 KH_2PO_4, 0.5 NaH_2PO_4, 1 $MgCl_2$, 11 D-glucose and 1.8 $CaCl_2$ (pH 7.4). Isolated arteries were cut into rings (~2 mm length) and used for wire myography or cell isolation.

For VSMC isolation, artery rings were cut open and incubated in dissociation medium (DM) containing 1.5 mg/mL papain (Sigma Aldrich, UK) for 1 h at 4°C. Composition of DM was (in mM): 110 NaCl, 5 KCl, 10 HEPES, 0.5 KH_2PO_4, 0.5 NaH_2PO_4, 10 $NaHCO_3$, 10 taurine, 0.5 EDTA, 10 D-glucose, 0.16 $CaCl_2$, 2 $MgCl_2$, 0.03 phenol red (pH 7.3). Dithiothreitol (0.5 mg/mL) was subsequently added to the medium to catalyse papain activity and incubated for 5 min at 37°C. The tissue pieces were then transferred into a new vial containing 0.4 mg/mL collagenase (type XI, Sigma Aldrich, UK) and incubated at 37°C for 4 min. The tissue pieces were subsequently washed twice in fresh DM and gently triturated with a smoothed glass Pasteur pipette.

Cell fluorescence imaging

Freshly isolated aortic, mesenteric and pulmonary artery VSMCs were placed on a glass-bottomed 35 mm petri dish and allowed to settle for 10 min prior to imaging. Individual VSMCs were imaged using a LSM510 META confocal laser scanning system (Zeiss, UK) connected to an inverted AxioVert 200 microscope with a 63X objective (Zeiss, UK) and controlled using the Zen 2012 (Blue edition) software (Zeiss, UK). To reveal expression and cellular localisation of the ChR2(H134R)-eYFP in isolated VSMCs, an excitation wavelength of 514 nm was used and an emission wavelength of 522 nm was recorded. All confocal images were acquired with identical settings. Cellular distribution of eYFP was estimated via fluorescence intensity plots of cross sections taken from these images. The fluorescence intensity of each cross sections was normalised for the maximal intensity measured within the same cross section.

Relative expression levels of ChR2(H134R)-eYFP in VSMCs isolated from ChR2(H134R)-SM mice were assessed using epifluorescence microscopy and quantified as the average fluorescence measured from at least 49 cells (from each vessel type) as outlined in (Adomaviciene *et al.*, 2013; McCloy *et al.*, 2014).

All epifluorescence images were acquired using identical settings with an AxioCam MRm camera (Zeiss, UK) connected to an Axio Vert.A1 inverted microscope with a 10X objective (Zeiss, UK), using Zen 2012 (Blue edition) software (Zeiss, UK). The fluorescence intensity of individual cells was corrected against the intensity point of the background (determined in a square box of 100 × 100 pixels in size outside the contour

of the cell) using the ImageJ software (National Institutes of Health, MD, USA) and expressed in arbitrary units. ImageJ was also used for figure preparation.

Electrophysiology

All patch-clamp current recordings were performed using an Axopatch 200A amplifier (Axon Instruments, CA, USA) controlled by GePulse software (http://users.ge.ibf.cnr.it/pusch/programs-mik.htm) with a 16 bit D-A and A-D converter (PCI-6221, National Instruments, UK). For whole-cell recordings of ChR2(H134R) currents in isolated VSMCs, the intracellular solution contained (in mM): 80 K-Aspartate, 50 KCl, 1 $MgCl_2$, 3 MgATP, 10 EGTA, 10 HEPES, 3 $CaCl_2$ and 10 NaCl (pH 7.4). The extracellular solution contained (in mM): 5.4 KCl, 140 NaCl, 1 $MgCl_2$, 1.8 $CaCl_2$, 10 HEPES and 10 D-glucose (pH 7.4). In perforated-patch current-clamp recordings of V_m, the intracellular solution was supplemented with amphotericin (0.3 mg/ml) and MgATP was removed. For whole-cell recordings of Ca_V currents, the intracellular solution contained (in mM): 112 CsCl, 3 Na_2-ATP, 5 HEPES, 10 EGTA and 1 $MgSO_4 \cdot 7H_2O$ (pH 7.4). The extracellular solution contained (in mM): 104 NaCl, 5.4 CsCl, 20 TEA-Cl, 5 $BaCl_2$, 1 $MgCl_2$, 1 NaH_2PO_4, 10 D-glucose and 5 HEPES (pH 7.4). Pipettes were pulled from borosilicate glass capillary tubes (Harvard Apparatus, UK) using a Narishige PC-10 pipette puller (Narishige, Japan). Pipette tip diameter yielded a resistance of 2-3 MΩ in the working solutions. The bath was grounded through an Ag/AgCl reference electrode. In whole-cell recordings, the series resistance was usually compensated to achieve a maximal effective series resistance lower than 5-10 MΩ. Experiments were conducted at 20-22°C. The cell capacitance was assessed by measuring the area under the capacitive transient elicited by a 10 mV voltage step or using the cell capacitance compensation circuit of the amplifier. Currents were filtered at 2 kHz and sampled at 10 kHz. Electrophysiology data were analysed with Igor Pro (Wavemetrics, OR, USA).

Myography

Isolated artery rings (~2 mm in length) were mounted on a DMT 410A wire myograph (Danish Myo Technology, Denmark) using tungsten wires (diameter 25-40 μm) and submerged in PSS solution at 37 °C (Mulvany and Warshaw, 1979; Tammaro *et al.*,

2004; Ward and Snetkov, 2004). In some experiments, $CaCl_2$ was not included in the PSS solution; this nominally Ca^{2+}-free solution was termed "Ca^{2+}-free PSS".

Artery rings were placed under a tension corresponding to 0.9 times the diameter of the vessel at 100 mmHg (aorta), 70 mmHg (mesenteric artery) or at 30 mmHg (pulmonary artery) (Mulvany and Warshaw, 1979; Ward and Snetkov, 2004). After an equilibration period of 40 min, the artery rings were exposed to a 50 mM increase in the extracellular KCl concentration until a maximal contractile response was observed. The vessels were then washed until tension returned to baseline and the exposure to high KCl was repeated until reproducible contractions were obtained. Following washout and return of tension to baseline, the artery rings were then exposed to periods of blue light or pharmacological agents, as indicated in the main text. Myography data were recorded with the Chart software (Dr Dempster, Strathclyde University) and analysed with Igor Pro (Wavemetrics, OR, USA).

pH imaging

Freshly isolated aortic VSMCs were AM-loaded with cSNARF1 (10 min) and imaged for pH ratiometrically (Zeiss LSM confocal system; excitation 555 nm, emission 640 and 580 nm) in an extracellular solution containing (in mM): 5.4 KCl, 140 NaCl, 1 $MgCl_2$, 1.8 $CaCl_2$, 10 HEPES and 10 D-glucose (pH 7.4). Fluorescence was measured before and after a 7 min exposures to blue light (470 nm) of various intensity, as indicated in the main text. The fluorescence ratio was converted to pH_i with a calibration curve determined in separate experiments using the protonophore nigericin (20 μM) to equilibrate intracellular and extracellular pH in intact aortic VSMCs superfused in high-K^+ solutions.

SUPPLEMENTARY FIGURES

Suppl. Fig. 1

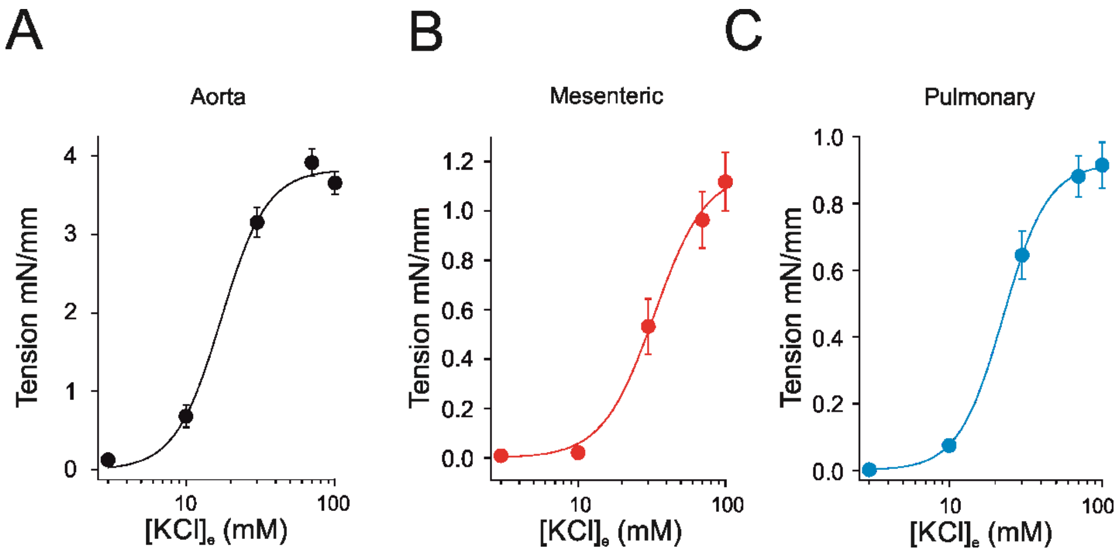

Supplementary Figure 1. *Effects of [KCl]$_e$ on the tension of rings of various artery types isolated from ChR2(H134R)-SM mice. Mean tension versus [KCl]$_e$ relationships for the different artery types (aortic,* ***A****, (N=20, n=32); mesenteric,* ***B****, (N=5, n=11) and pulmonary,* ***C****, (N=5, n=11)). The curves through the filled symbols represent the best fit of the data with Eq. 5.*

Suppl. Fig. 2

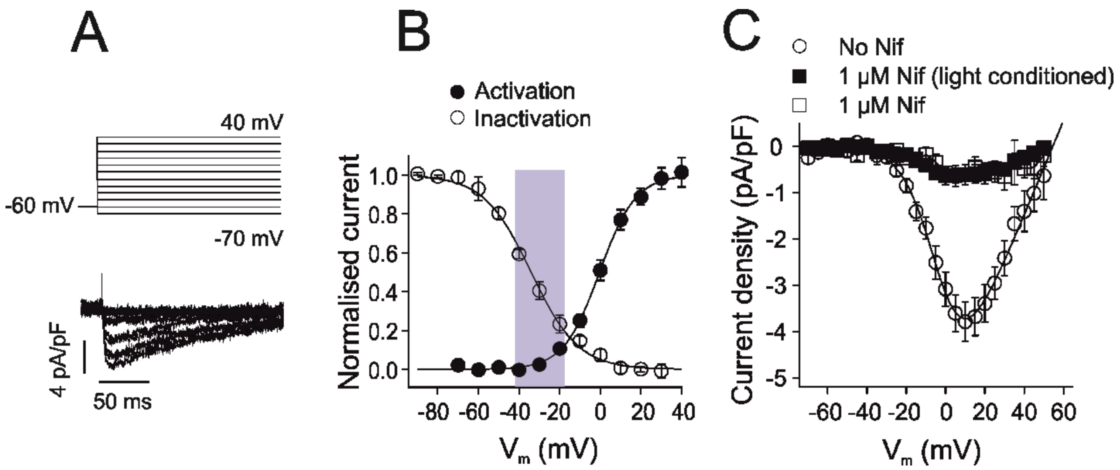

Supplementary Figure S2. *Ca_V channel currents in isolated aortic VSMCs* ***A****. Representative whole-cell Ca_V channel currents obtained from aortic VSMCs isolated from ChR2(H134R)-SM mice in response to the stimulation protocol shown in the top panel. For clarity, only the currents elicited every 20 mV are displayed.* ***B****. Mean steady-state activation (N=8, n=15) and inactivation (N=7, n=12) curves for Ca_V channels measured in aortic VSMCs. Smooth curves represent the fit of the data using Eq. 3 and 4 (activation and inactivation, respectively). Grey bar highlights the range of V_m that intact VSMCs can experience as a result of ChR2(H134R)-mediated depolarisations.* ***C****. Mean whole-cell Ca_V currents versus V_m relationships in the absence (N=8, n=15) or presence of 1 µM nifedipine (Nif) which was either kept in the dark (N=5, n=9) or exposed to blue light (2.4 mW/mm^2) for 10 min (light conditioned) (N=6, n=20), as indicated. Curves through symbols represent the best fit of the data with Eq. 3.*

Suppl. Fig. 3

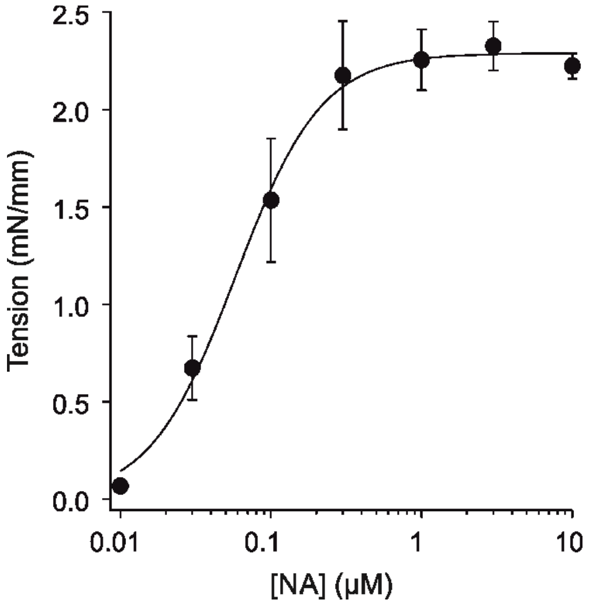

Supplementary Figure 3. *Effects of noradrenaline on the tension of aortic rings isolated from ChR2(H134R)-SM mice. Mean tension versus noradrenaline concentration ([NA]) relationship (N=5, n=8). The curve through the filled symbols represent the best fit of the data with Eq. 5.*

TABLES

Supplementary Table 1. *Parameters for tension versus $[KCl]_e$ relationships for artery rings obtained from ChR2(H134R)-SM mice.*

	ChR2(H134R)-SM mice		
	**Aorta**	**Mesenteric artery**	**Pulmonary artery**
***EC_{50}* (mM)**	18.2±1.3 (N=20, n=32)	39.0±8.5 (N=5, n=11)	23.2±1.5 (N=5, n=11)
s	3.3± 0.2 (N=20, n=32)	2.9±0.4 (N=5, n=11)	3.1±0.2 (N=5, n=11)
***T_{ss}* (mN/mm)**	4.0±0.2 N=20, n=32)	1.3±0.1 (N=5, n=11)	0.9±0.1 (N=5, n=11)

Parameters ($EC_{50,}$ s and T_{ss}) obtained from the Hill fit (Eq. 5) of the tension versus $[KCl]_e$ relationship for aortic, mesenteric and pulmonary artery rings obtained from ChR2(H134R)-SM mice.

Supplementary Table 2. *Parameters for tension versus [NA] relationships for artery rings obtained from ChR2(H134R)-SM mice.*

	ChR2(H134R)-SM mice
	**Aorta**
***EC_{50}* (mM)**	0.1±0.1 (N=5, n=8)
s	1.9± 0.3 (N=5, n=8)
***T_{ss}* (mN/mm)**	2.4±0.1 (N=5, n=8)

Parameters ($EC_{50,}$ s and T_{ss}) obtained from the Hill fit (Eq. 5) of the tension versus [NA] relationships for aortic rings obtained from ChR2(H134R)-SM mice.

REFERENCE LIST

Adomaviciene, A. *et al.* (2013) 'Putative pore-loops of TMEM16/anoctamin channels affect channel density in cell membranes.', *The Journal of physiology*, 591(Pt 14), pp. 3487–505.

McCloy, R. A. *et al.* (2014) 'Partial inhibition of Cdk1 in G2 phase overrides the SAC and decouples mitotic events', *Cell Cycle*, 13(9), pp. 1400–1412.

Mulvany, M. J. and Warshaw, D. M. (1979) 'The active tension-length curve of vascular smooth muscle related to its cellular components.', *The Journal of general physiology*, 74(1), pp. 85–104.

Sambrook, J. and Russell, D. W. (2001) *Molecular Cloning: A Laboratory Manual*. Cold Spring Harbor Laboratory Press (Molecular Cloning: A Laboratory Manual).

Tammaro, P. *et al.* (2004) 'Pharmacological evidence for a key role of voltage-gated K^+ channels in the function of rat aortic smooth muscle cells.', *British journal of pharmacology*, 143(2), pp. 303–17.

Ward, J. P. T. and Snetkov, V. A. (2004) 'Determination of signaling pathways responsible for hypoxic pulmonary vasoconstriction: use of the small vessel myograph.', *Methods in enzymology*, 381, pp. 71–87.
